# Dynamic matrix engineering promotes nascent protein deposition to drive cell migration and expedite Re-epithelization in chronic wound

**DOI:** 10.1016/j.bioactmat.2025.10.020

**Published:** 2025-10-28

**Authors:** Songsong Shi, Wei Zhang, Yuanman Yu, Jiaqi Qiu, Runzhi Huang, Shizhao Ji, Xue Qu

**Affiliations:** a Key Laboratory for Ultrafine Materials of Ministry of Education, Frontiers Science Center for Materiobiology and Dynamic Chemistry, School of Materials Science and Engineering, East China University of Science and Technology, Shanghai, 200237, China; b Department of Burn Surgery, The First Affiliated Hospital of Naval Medical University, Shanghai, 200433, China; c Shanghai Frontier Science Research Base of Optogenetic Techniques for Cell Metabolism, East China University of Science and Technology, Shanghai, 200237, China; d Wenzhou Key Laboratory of Tissue Regeneration Medical Materials, Wenzhou, 325000, China

**Keywords:** Hydrogel, Stress relaxation, Cell migration, Nascent protein, Chronic wound healing

## Abstract

Chronic wound healing remains a formidable clinical challenge, fundamentally hindered by stalled re-epithelialization caused by dysfunctional cell migration arising from a disordered mechano-biochemical microenvironment. Current **therapeutic** strategies relying on externally-assisted growth factors or mechanical stimulation often neglect the inherent capacity of the native, dynamic extracellular matrix (ECM) to govern cell behavior, specifically its viscoelasticity. By engineering a reversible hydrazone-crosslinked lysozyme-polyethylene glycol (LZM-PEG) dynamic hydrogel, we elucidated the mechanism whereby enhanced network dynamics activate early cell mechanotransduction via the integrin-FAK signaling axis, promoting nascent protein deposition which subsequently drives directed cell migration. Importantly, this mechano-biological effect exhibits distinct network dynamics dependence, as evidenced by the complete abolition of cell migration upon network rigidification, suggesting that matrix network dynamics constitutes a key regulatory factor. Diabetic mouse models demonstrated that this dynamic hydrogel accelerates chronic wound re-epithelialization by driving epithelial cell migration, solely by recapitulating ECM dynamics without exogenous interventions. This therapeutic effect reveals that the intrinsic mechano-bioactivity embedded in hydrogel's dynamic network can accelerate tissue repair by modulating *in situ* cell behavior. Collectively, this study uncovers a mechano-biological axis: matrix dynamics-nascent protein deposition-cell migration, which provides mechanobiological insights into tissue repair, and offers a novel “materiobiology” design strategy for next-generation regenerative materials.

## Highlights

• A reversible hydrazone-crosslinked LZM-PEG hydrogel was engineered with independently tunable viscoelasticity to mimic ECM-like dynamic mechanical behavior.
• Dynamic network behavior activates integrin–FAK mechanotransduction, triggering nascent protein deposition and directional cell migration.
• Matrix rigidification abolishes migration, highlighting hydrogel network dynamics as a key regulator of epithelial cell behavior.
• The dynamic hydrogel promotes re-epithelialization in diabetic wounds without exogenous biochemical or physical stimuli.
• This study reveals a matrix dynamics–protein deposition–cell migration axis, offering a materiobiological strategy for regenerative material design.

## Graphical abstract

**Figure:**
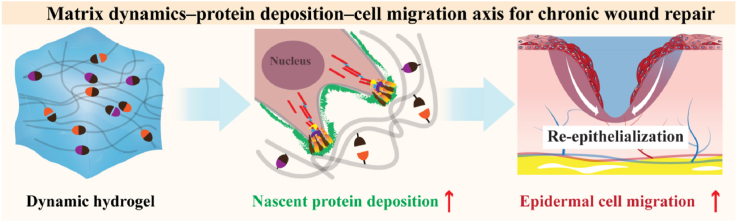

## 1 Introduction

Cell migration plays a pivotal role in development, homeostasis, immune cell trafficking, and wound healing. Skin wound healing requires rapid re-epithelialization to restore a continuous and functional epidermal barrier, which protects the organism from infection [1,2]. Re-epithelialization predominantly depends on the migration of epithelial cells from the wound edges to the wound bed [3,4]. However, the complex pathological microenvironment of diabetic skin wounds—characterized by tissue sclerosis, accumulation of advanced glycation end-products (AGEs), abnormal growth factor expression, and an imbalance between matrix metalloproteinases (MMPs) and tissue inhibitors of metalloproteinases (TIMPs)—disrupts ECM deposition and cell-ECM interactions [[5], [6], [7], [8]]. This disruption limits the availability of adhesion sites and physical space required for cell migration, impairs the cells' ability to respond to migration signals from the ECM, and ultimately leads to dysfunctional cell migration and impaired re-epithelialization [9,10].

Substantial research has highlighted that activation of mechanotransduction pathways in skin cells can effectively regulate cell behavior and accelerate wound healing [[11], [12], [13]]. However, these strategies generally require the application of exogenous force fields (e.g., magnetic fields, light fields, sound fields*, etc.*) and have not achieved sustained *in situ* regulation of cells at the wound margins. Tissues and ECM at different developmental stages exhibit dynamic mechanical behaviors, including changes in stiffness and time-dependent stress relaxation [14,15]. ECM biomolecule networks store force signals and gradually release energy that causes network deformation in a time-dependent manner [[16], [17], [18]]. This network dynamics can mechanically regulate cell behavior, disease progression, and tissue development [[19], [20], [21]]. By mimicking this biological environment, dynamic hydrogels with stress relaxation properties have been designed as artificial ECM to activate mechanotransduction and regulate cell behavior [3,22].

However, existing studies predominantly concentrate on the unidirectional regulation of cell fate by the inherent properties of materials and overlook the feedback from cells to the material by autonomously constructing the surrounding extracellular microenvironment. Therefore, these studies are insufficient to fully elucidate the bidirectional signal transduction between the matrix and cells, as well as the upstream and downstream regulatory mechanisms of cell behavior mediated by three-dimensional (3D) artifical network dynamics. Moreover, the *in situ* repair of tissue structure *in vivo*, enabled by the dynamic characteristics of artificial ECM has not yet been explored sufficiently. This oversight can be attributed to the fact that dynamic hydrogels are only used as excellent delivery carriers for trauma treatment [23,24].

**Scheme 1:**
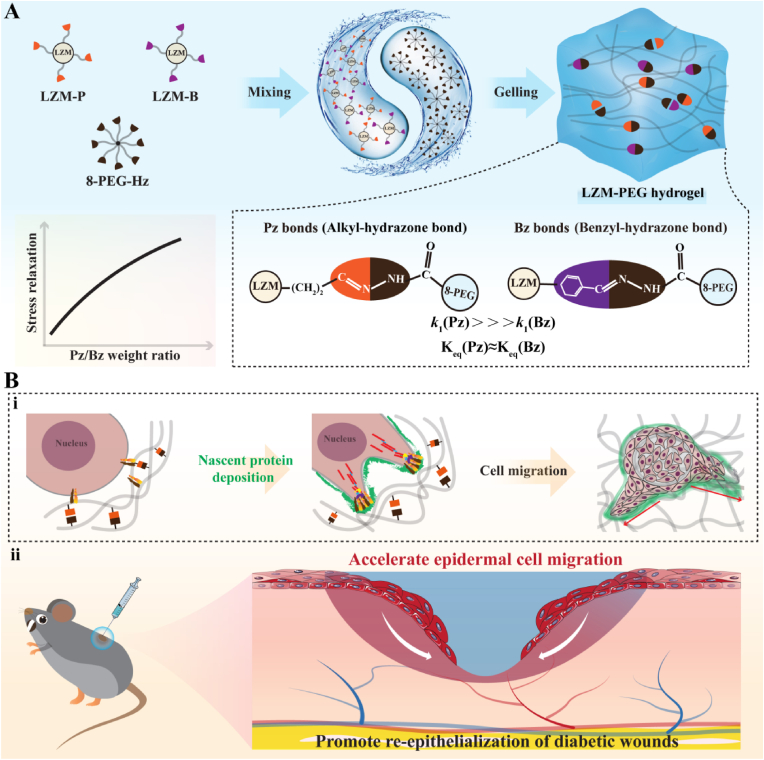
Schematic diagram for the formation of LZM-PEG hydrogel and its application for chronic skin wound healing. (A) Schematic diagram for the formation of LZM-PEG hydrogel. (B) i: Hydrogel network dynamics promote nascent protein deposition and accelerate cell migration. ii: LZM-PEG hydrogel with enhanced network dynamics effectively translate in vitro pro-cell migration effects into accelerated slow wound reepithelialization and wound healing.

Drawing on the principles of materiobiology and research into mechanical regulation of wound healing [25,26], we bionically designed a hydrogel-based dynamic material to investigate how cellular feedback modulates cell-hydrogel interactions and confirmed the translation of the powerful cellular response of the hydrogel viscoelasticity in vitro to an accelerated chronic wound healing response *in vivo*. In this study, we designed LZM-PEG crosslinked by reversible hydrazone bonds, with network dynamics controlled by the exchange kinetics of these bonds (Scheme 1A). Our experimental findings demonstrate that hydrogels with enhanced network dynamics effectively activate early mechanosensing pathways and promote nascent protein secretion and subsequent deposition in hydrogels, providing more anchor points for cells to migrate into or permeate the hydrogel. Furthermore, in diabetic mice model with full-thickness excisional wounds, we show that LZM-PEG hydrogels with enhanced network dynamics modulate cell adhesion and cytoskeletal reorganization during the early stages of wound healing, promoting accelerated epithelial cell migration and re-epithelialization (Scheme 1B). This work introduces a promising materiobiological strategy that utilizes intrinsic material dynamics to sustain mechanochemical signaling loops between cells and the ECM, overcoming the negative regulatory effects of pathological factors in the chronic wound microenvironment and directly activating cell migration. The regulation of hydrogel network dynamics represents a crucial target for the design of next-generation active materials for tissue repair and regeneration.

## 2 Results

### 2.1 LZM-PEG hydrogel network dynamics depends on the relative weight ratio of alkyl-hydrazones to benzyl-hydrazones

**Fig. 1:**
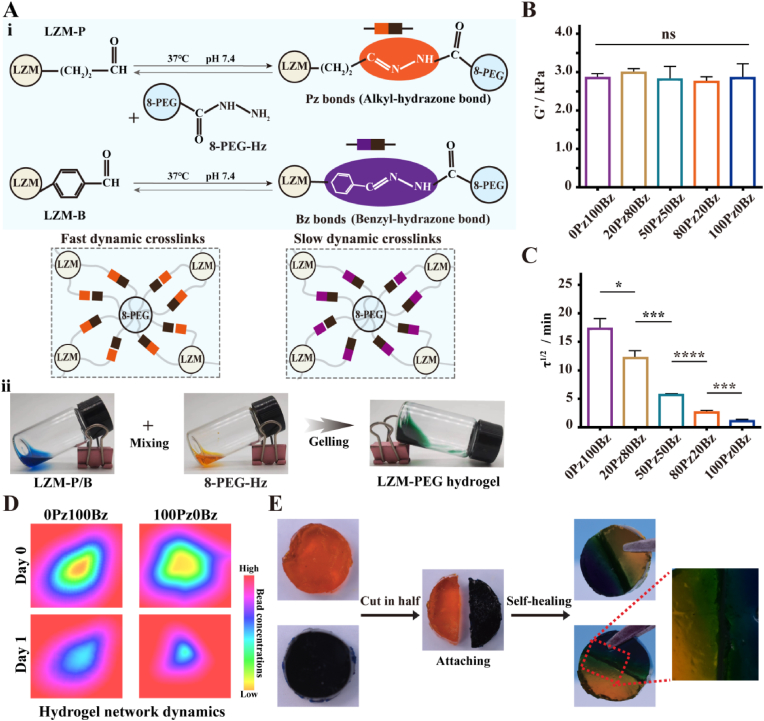
LZM-PEG hydrogels crosslinked by different formulations of alkyl-hydrazone bonds (Pz) or benzyl-hydrazone bonds (Bz) have different network dynamics. (A) i:Schematic illustration of the crosslinking between LZM-P (LZM-B) and eight-armed PEG terminally modified hydrazide via Pz bonds (Bzbonds; ii: Representative optical pictures for preparing LZM-PEG hydrogel. (B) Rheological test shows all hydrogels have the same elastic modulus (G′) (n = 3). (C) Stress relaxation test shows increasing the fraction of Pz while decreasing that of Bz enhances hydrogel network dynamics. τ^1/2^ was used to characterize hydrogel network dynamics.(n = 3) (D) Representative heat map of matrix deformation over 1 day as determined by the tracking of embedded fluorescent bead displacements. (E) Photographic evidence demonstrating the self-healing ability of LZM-PEG, where broken hydrogels regenerate bonding, as illustrated in the inset showing the regeneration process at the fracture site. All data are presented as mean ± SD. Statistical significance was calculated by unpaired two-tailed Student's *t*-test and Ordinary one-way ANOVA. ∗P < 0.05,∗∗P < 0.01, ∗∗∗P < 0.001, and ∗∗∗∗P < 0.0001. (100Pz0Bz, 80Pz20Bz, 50Pz50Bz, 20Pz80Bz and 0Pz100Bz were prepared by mixing Pz and Bz at the weight ratio of 100 %:0 %, 80 %:20 %, 50 %:50 %, 20 %:80 % or 0 %:100 %, respectively.)

We prepared a series of dynamic hydrogels through reversible covalent bonds between eight-armed PEG-hydrazide and LZM-aldehyde (P, propionaldehyde; B, benzaldehyde) to yield hydrazone bonds (alkyl-hydrazone (Pz) and benzyl-hydrazone (Bz)) (Fig. 1A, Fig. S1). By precisely adjusting the weight ratio of Pz and Bz in the hydrogel backbone, the hydrogel network dynamics were customized without affecting the initial elastic modulus (∼3 kPa) (Fig. 1B). Stress relaxation rate is quantified by the time required for the normalized stress to dissipate by half (τ_1/2_) to characterize hydrogel network dynamics. As hypothesized, the 100Pz0Bz hydrogels exhibited the fastest τ_1/2_ (∼65 s), whereas 0Pz100Bz hydrogels exhibited the slowest τ_1/2_ (∼1042 s) (Fig. 1C). By tracking the distribution of fluorescent beads embedded within hydrogel, we verified that beads in 100Pz0Bz hydrogel more easily migrate towards the center during continuous cell movement in comparison to 0Pz100Bz hydrogel (Fig. 1D), implying that cell-hydrogel interactions in 100Pz0Bz hydrogels are more likely to occur. These results indicate that increasing the fraction of Pz while simultaneously decreasing that of Bz enhances hydrogel network dynamics without affecting hydrogel stiffness. This can be attributed to the fact that the two types of hydrazone bonds have drastically different kinetic binding constants (*k*_1_, *k*_2_) yet similar equilibrium binding constants (*K*_eq_) [27,28]. Specifically, the former determines the bond exchange rate and dynamics within the hydrogel network, whereas the latter governs the crosslink density and macroscopic mechanical properties of hydrogel network. Small molecule kinetic studies have demonstrated that Pz possesses over several orders of magnitude larger kinetic binding constants than Bz, resulting in a substantially slower stress relaxation behavior for Bz crosslinked hydrogels relative to Pz crosslinked hydrogels [29]. An identical amount of 4-amino-DL-phenylalanine was incorporated into hydrogels to accelerate the exchange rate of hydrazone bonds as well as the stress relaxation behavior of hydrogels without causing any difference in the concentrations of the hydrogel backbones [30,31]. To examine whether diverse hydrogel formulations can affect hydrogel stability, all hydrogels were immersed in cell medium at 37 °C. The results showed no difference in hydrogel swelling after 3 days, except for the disintegration of 100Pz0Bz hydrogel (Fig. S2). Self-healing tests showed that fractured hydrogels can reintegrate due to the reversible hydrazone bonds, enabling hydrogels to maintain *in situ* formation and integrity in practice (Fig. 1E).

We next investigated the viability of keratinocyte (HaCat) and fibroblast (L929) in hydrogels. Both live-dead staining and lactate dehydrogenase (LDH) assay revealed that cells are more proliferative in the hydrogels with enhanced network dynamics (80Pz20Bz) (Fig. S3). To determine whether differences in cell viability were influenced by variations in nutrient diffusion rates within the hydrogels, we co-incubated the hydrogels with Rhodamine B and assessed fluorescence distribution. Our results confirmed that nutrient diffusion was consistent across hydrogels with varying network dynamics (Fig. S4). We hypothesize that the differences in hydrogel network dynamics, arising from the selective use of reversible hydrazone bonds, enable the material to respond to cell forces, thus providing a 3D mechanical microenvironment that is adapted to cell behavior.

### 2.2 LZM-PEG hydrogels with enhanced network dynamics promotes cell migration

Our previous work has demonstrated that LZM can provide cell adhesion sites to hydrogels on account of its RGD-like tripeptide sequence [31,32]. By immunofluorescent labelling and 3D reconstruction, we observed that L929 cell clusters on LZM-modified hydrogels were able to spread across the hydrogel surface and gradually infiltrate into the hydrogel interior. Nevertheless, such behavior were not witnessed on BSA hydrogels (Fig. S5A and B). Given the sole disparity in backbone composition between the two hydrogels, we ascribe the variation in cell performance to the recognition and binding of RGD by the cells. We then probed into the impact of hydrogel network dynamics on single-cell spreading. Sample spreading cells on 80Pz20Bz hydrogels began to sprout after 3 h and thereafter exhibited a low aspect ratio. In contrast, cells on 0Pz100Bz hydrogels required more time for sprouting and morphological changes (Figs. S5C,D,E). Thus, these data suggest that enchanced hydrogel network dynamics, in combination with LZM may activate subsequent biological events by mediating cell adhesion and cytoskeletal remodeling.

**Fig. 2:**
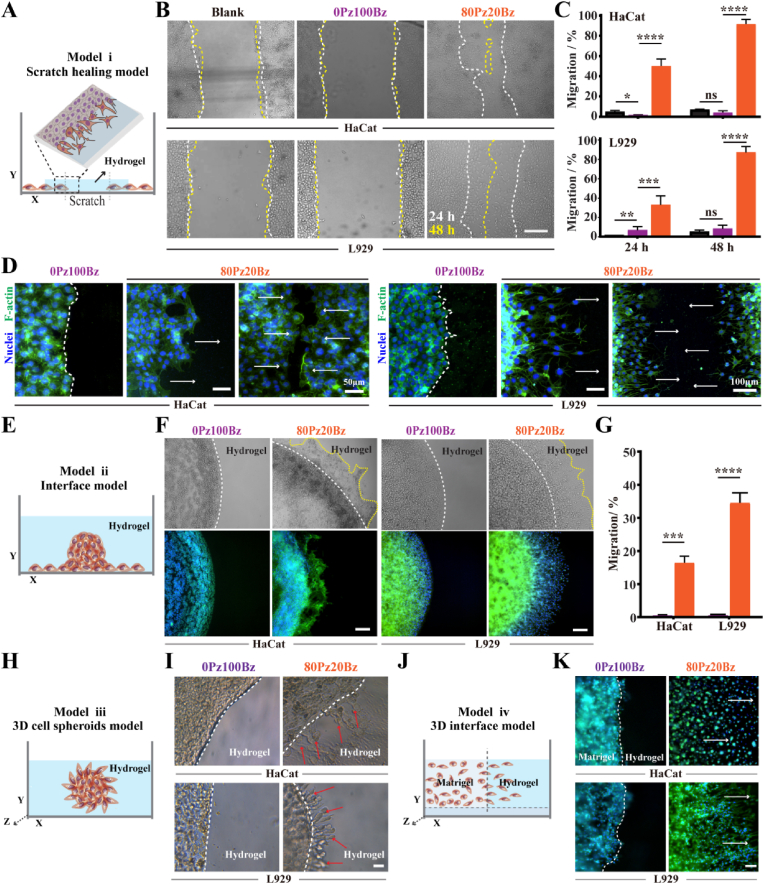
LZM-PEG hydrogels with enhanced network dynamics promotes cell migration. (A) Schematic illustration of model **i** migration assays. (B) Photograph evidences suggest that hydrogels with enchanced network dynamics promote scratch healing (Scale bars: 200 μm) (C) Quantification of scratch healing (n = 3). (D)Representative fluorescent images of migrating cells in model **i** (Scale bar: 50 μm) (E) Schematic illustration of model **ii** migration assays. (F) Representative images of migrating HaCat and L929 in model **ii**. (Scale bar: 100 μm) (n = 3) (G) Quantification of migrating HaCat and L929 in model **ii**. (H) Schematic illustration of model **iii** migration assays. (I) Representative images of migrating cells in model **iii**. (Scale bar: 50 μm) (J) Schematic illustration of model **iv** migration assays. (K) Representative images and quantification of migrating cells in model **iv.**(Scale bar: 100 μm). (n = 3). All results indicate that cells are more likely to migrate into highly dynamic hydrogels. All data are presented as mean ± SD. Statistical significance was calculated by unpaired two-tailed Student's *t*-test. ∗P < 0.05,∗∗P < 0.01, ∗∗∗P < 0.001, and ∗∗∗∗P < 0.0001.

To test how tissue organization influences cell migration, we devised cell migration modelswith varying hydrogel spaces. First, the modified scratch healing model, in which scratches were filled with hydrogels, simulated the cell migration process in a wound (Fig. 2A). Second, the interface model enabled cell migration from spheroids along the interface between the Petri dish substrate and hydrogels (Fig. 2E). The lack of crosslinking between the top and bottom resulted in a lack of lateral confinement, permitting the continuous cell migration driven by the hydrogel. Third, hydrogels encapsulating cell spheroidsenabled the direct characterization of 3D cell migration (Fig. 2H). Lastly, a 3D traversing interface model was created between Matrigel and hydrogels to mimic cell migration from the wound edge into the hydrogels (Fig. 2J).

**Fig. 3:**
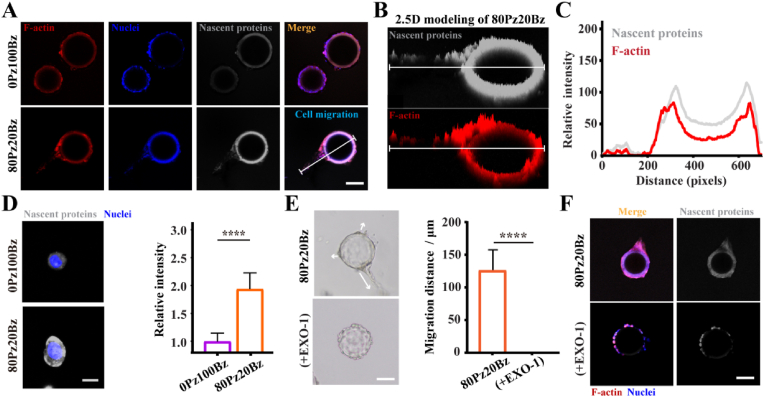
Nascent protein deposition regulates HaCat cell migration in LZM-PEG hydrogel. (A) Representative fluorescent images showing HaCat cell migration in low-dynamic and high-dynamic groups. (Scale bar: 100 μm) (B) 2.5D modeling of fluorescent images depicting HaCat cell migration in 80Pz20Bz hydrogels. (C) Co-localization curves of nascent proteins (gray) and F-actin (red) in HaCat cells migrating within 80Pz20Bz, illustrating the correlation between nascent protein deposition and cell migration. (D) Representative fluorescent images and quantitative analysis of nascent protein intensity in HaCat cells after early culture, demonstrating that dynamic hydrogel environments promote nascent protein secretion. (Scale bar: 10 μm) (n = 5). (E) Representative bright-field images and quantification of HaCat cell migration, showing suppressed migration in 80Pz20Bz when cell treated with EXO-1 (nascent protein inhibitor). (Scale bar: 100 μm) (n = 3). (F) Fluorescent images of HaCat cells treated with EXO-1, which inhibits nascent protein deposition and abolishes cell migration in 80Pz20Bz. (Scale bar: 100 μm) All data are presented as mean ± SD. Statistical significance was calculated by unpaired two-tailed Student's *t*-test.∗∗∗∗P < 0.0001.

HaCat and L929 cells were utilized in these models, with the former enabling for collective cell migration and the latter performing single-cell migration [3,33]. Sample migrating cells showed that enhanced network dynamics promotes cell migration irrespective of the applied model. In scratch healing model (i), both types of cells within 80Pz20Bz hydrogels migrated towards the scratch center and nearly closed the scratch after 48 h. In contrast, this phenomenon was not observed in blank group and 0Pz100Bz hydrogel (Fig. 2B and C). HaCat cells exhibited sheet-like collective migration driven by 80Pz20Bz hydrogel, while L929 cells displayed single-cell migration (Fig. 2D). Meanwhile, RT-qPCR results revealed that the migration-related markers in HaCat cells (*YAP1*, *ITGB1,* and *CDH2*) and L929 cells (*YAP1*, *ITGB1,* and *RAC1*) were significantly up-regulated in 80Pz20Bz hydrogel compared with those in 0Pz100Bz hydrogel (Fig. S6A and B). No cell migration wae observed when the 80Pz20Bz hydrogels was replaced with the hydrogel degradation solution of the same composition and concentration (Fig. S6C). These results suggest that the continuous cell migration is caused by cell-hydrogel interactions rather than hydrogel degradation products. Similar tocell migrationobservedin model (i), we observed significant cell migration encapsulatedin 80Pz20Bz hydrogelsin the interface model (ii) (Fig. 2F and G), 3D cell spheroids model(iii) (Fig. 2I) and 3D traversing interface model (iv) (Fig. 2K). In contrast, cells in 0Pz100Bz hydrogels did not get rid of cell clusters into the hydrogel. Notably, a clearly visible demarcation line was directly observed between the cell/cell clusters and 0Pz100Bz hydrogels, irrespective of the model used, implying that there is resistance at the cell-hydrogel interface limiting cell migration. Collectively, these results suggest that LZM-PEG hydrogels with enhanced network dynamics are more favorable for both collective and single-cell migration. whereas slow stress-relaxed hydrogels network physically restrict the movement ofcell/cell clusters.

### 2.3 Nascent protein deposition regulated bystrong hydrogel network dynamics promotes cell migration

To elucidate the critical mechanisms by which the enhanced dynamicity of LZM-PEG hydrogels regulates cell migration, we encapsulated dextran microspheres coated with HaCaT cells within the hydrogels, enabling more precise observation of the hydrogel's impact on cell migration at a single-cell level. The results demonstrated that in the 80Pz20Bz hydrogel, HaCaT cells exhibited significant and active migratory behavior, whereas no noticeable migration was observed in the 0Pz100Bz hydrogel (Fig. S7A). Given that cell migration is regulated by ECM proteins secreted by the cells themselves, we employed a fluorescence labeling technique based on a click chemistry reaction between azide-modified histidine-alanine (AHA, a methionine analog) and a fluorescently-labeled cyclooctyne (DBCO-488) to visualize and investigate changes in nascent protein deposition during the cell migration process [27,34]. AHA are incorporated into proteins as they are synthesized, and can be labeled with DBCO-488, thereby rendering nascent proteins fluorescent. The results indicated that in the 80Pz20Bz hydrogel, active cell migration was accompanied by extensive deposition of nascent proteins, while in the 0Pz100Bz hydrogel, nascent protein generation was nearly undetectable (Fig. 3A). Subsequent fluorescence co-localization analyses revealed that the deposition of nascent proteins in the 80Pz20Bz hydrogel exhibited a spatiotemporal distribution consistent with migrating cells (Fig. 3B and C). These findings suggest that the interaction between cells and the hydrogel matrix primarily governs nascent protein deposition, playing a pivotal role in cell migration.

To further clarify the critical role of nascent protein deposition in regulating cell migration within the 80Pz20Bz hydrogel, we introduced Exo-1 to specifically inhibit the transport and secretion of ECM proteins [35]. We first demonstrated that enhanced hydrogel dynamics promot nascent protein secretion from the single-cell level after a 4-h culture period (Fig. 3D). However, following Exo-1 inhibition, cells retained a rounded morphology and exhibited significantly impaired migration (Fig. 3E,Fig, S7B). As expected, the migratory behavior of cells in the 80Pz20Bz hydrogel exhibited a positive correlation with nascent protein deposition. Together, these findings suggest that cell migration within engineered hydrogels depends on the interplay between nascent protein deposition and the dynamic properties of the hydrogel network, which facilitating the formation of new migratory pathways.

**Fig. 4:**
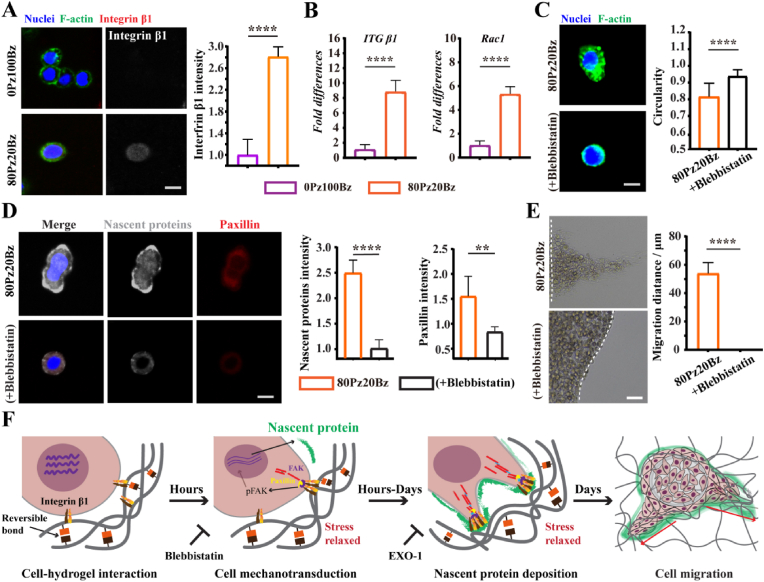
Cell mechanosensing in HaCat cells is activated by hydrogel network dynamics, promoting nascent protein secretion and cell migration. (A) Representative fluorescent images showing *Itgβ1* expression and its quantification in HaCat in different hydrogel groups. (Scale bar: 10 μm) (n = 8) (B) qRT-PCR analysis of *Rac1* and *Itgβ1* expression in L929 cells, showing upregulation of *Itgβ1* in strongly dynamic hydrogels. (n = 3) (C) Representative fluorescent images and quantification of cell circularity, demonstrating altered cell morphology in 80Pz20Bz when using blebbistatin (integrin clustering inhibitor). (Scale bar: 10 μm) (n = 18) (D) Representative fluorescent images and quantification of nascent protein deposition and paxillin intensity in HaCat cells, showing that early cell mechanotransduction activates nascent protein secretion. (Scale bar: 10 μm) (n = 5) (E) Bright-field images of HaCat cell migration in 80Pz20Bz, showing the inhibition of migration by blebbistatin treatment. (Scale bar: 100 μm) (n = 3) (F) Schematic illustration of enhanced hydrogel network dynamics for accelerated cell migration. The dynamic hydrogel network facilitates the formation of focal adhesions (FAs) and integrin clustering, leading to nascent protein secretion and remodeling, and epidermal cell migration. All data are presented as mean ± SD. Statistical significance was calculated by unpaired two-tailed Student's *t*-test. ∗∗P < 0.01, ∗∗∗P < 0.001, and ∗∗∗∗P < 0.0001.

To elucidate how the stress relaxation behavior of hydrogels influences nascent proteins secretion by target cells, we first characterized the cell morphology in hydrogels with varying stress relaxation after 4 h of incubation because subsequent cell behavior in hydrogels is tightly linked to early spreading morphology. Quantification of circularity revealed strong differences in cell spreading between cells in 80Pz20Bz hydrogels and cells in 0Pz100Bz hydrogels, suggesting that cells are more likely to engage in morphological changes when stimulated by enhanced network dynamics **(**Fig. S8A). Previous studies have demonstrated that cells initiate focal adhesion (FA) formation and mechanotransduction via integrin interactions with the hydrogel network [22,36]. The initial binding of integrins to RGD adhesion motifs promotes the activation of focal adhesion kinase (FAK) and the recruitment of paxillin, leading to integrin clustering and the formation of larger, more stable FAs [35]. Given the high stress relaxation characteristics of the 80Pz20Bz hydrogels, we observed upregulated *ITGB1* and *RAC1* expression in migrating cells (Fig. 4A and B). The increased *ITGB1* expression reflects a heightened capacity for mechanical sensing through the extracellular microenvironment. Concurrently, elevated *RAC1* level indicates enhanced formation of lamellipodial protrusions, which facilitates the exploration of the surrounding microenvironment by cells. Together, these results suggest that the soft, relaxative hydrogel promotes integrin-mediated mechanotransduction and activates cytoskeletal reorganization, enabling cells to dynamically probe and adapt to substrate mechanics during migration. Subsequently, we examined cell adhesion in hydrogels with enhanced network dynamics, which are known to facilitate cell spreading and migration. Cells within the 80Pz20Bz hydrogel exhibited peripheral paxillin structures, indicative of robust cell adhesion (Fig. S8B). In contrast, cells in the 0Pz100Bz hydrogel failed to form paxillin structures. Quantitative analysis revealed a significant increase in FAK activation in the 80Pz20Bz hydrogel, as evidenced by the overall intensity of FAK and phosphorylated FAK (pFAK) (Fig. S8C and D).

To further investigate the impact of hydrogel network dynamics on the initiation and progression of cell migration through contractility-mediated integrin clustering, we treated cells embedded in 80Pz20Bz hydrogel with blebbistatin, a well-established inhibitor of myosin-II ATPase activity and cell contractility, which is known to prevent the aggregation of distant integrin clusters [37,38]. Blebbistatin treatment decreased cell circularity and reduced paxillin structure formation, indicating that inhibition of cellular contractile forces significantly affects cell adhesion and spreading (Fig. 4C). To determine whether hydrogel dynamics influence integrin clustering and consequently affect nascent protein secretion and their intrinsic relationships, we further treated the cells with blebbistatin while simultaneously examining nascent protein deposition and paxillin expression. Immunofluorescence results demonstrated that blebbistatin inhibited the promotion of nascent protein secretion by 80Pz20Bz hydrogels (Fig. 4D). Moreover, the inhibition of integrin clustering by blebbistatin abolished the migratory effect of 80Pz20Bz hydrogels (Fig. 4E), suggesting that cellular mechanosensing directly influences hydrogel dynamic-mediated cell migration. Together, these findings indicate that 80Pz20Bz hydrogels enhance cell-matrix interactions and mediate cell migration by promoting integrin clustering, which activates downstream signaling pathways including paxillin and FAK expression, leading to increased nascent protein deposition.

*Early interactions between cells and hydrogels, along with* cell contraction-mediated cell adhesion promote integrin aggregation and activation of downstream signaling pathways. The stress-relaxed hydrogel network adapts to nascent protein secretion and deposition through cell contraction-mediated network rearrangements, leading to cellular morphological changes and migration (Fig. 4F). Hydrogel network dynamics is thereby a key mediator of encapsulated cell fate. Weakly dynamic hydrogels physically confine cell contractility and integrin clustering, causing the inhibition of cell signaling and migration.

### 2.4 LZM-PEG hydrogels with enhanced network dynamics accelerates chronic wound healing by promoting re-epithelialization

**Fig. 5:**
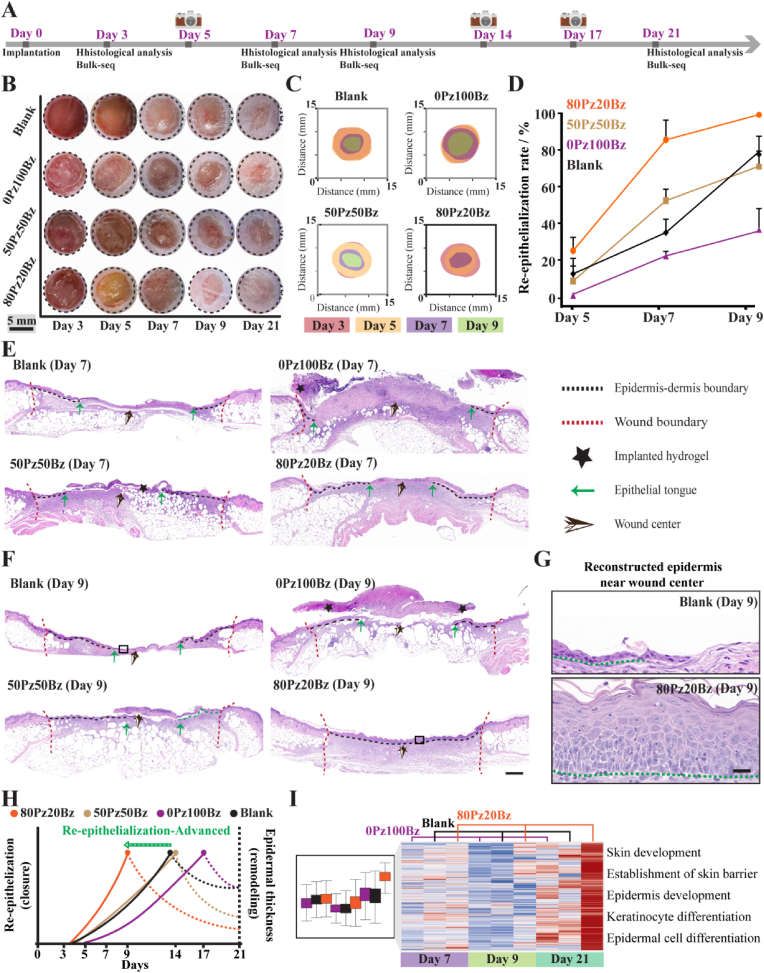
LZM-PEG hydrogels with enhanced network dynamics accelerates chronic wound healing by promoting re-epithelialization. (A) Schematic illustration of the experimental design for evaluating the therapeutic effects of LZM-PEG hydrogels in a db/db mouse model. (B) Representative images of wound healing from different groups on days 3, 5, 7, 9, and 21 after operation. (C) Schematic illustration of re-epithelialization in wounds from different groups on days 3, 5, 7, and 9 after operation. Each colored block represents the remaining non-re-epithelialized area at the corresponding point. (D) Corresponding analysis of reepithelialized area (n = 5). (E) Representative H&E images at day7.(Scale bar: 500 μm) (F) Representative H&E images at day9showing superior wound healing in the strongly dynamic hydrogel group compared to other groups.(Scale bar: 500 μm) (G) Reconstructed epidermis near wound center indicating that the regenerated epidermis in the 80Pz20Bz group is more systematic and mature. (H) Schematic timeline highlighting the processes of re-epithelialization and matrix remodeling in the different groups. (I) Bulk-RNA sequencing analysis of 0Pz100Bz versus blank versus 80Pz20Bz groups (n = 3 for each group). Heatmap (left) showing hierarchical clustering of differentially expressed genes (p value < 0.05& |log2FC| > 1) between three groups, and corresponding gene set enrichment analysis showing the enriched terms in 0Pz100Bz versus blank versus 80Pz20Bz groups. All data are presented as mean ± SD. Statistical significance was calculated by unpaired two-tailed Student's *t*-test and Ordinary one-way ANOVA. ∗∗∗P < 0.001, and ∗∗∗∗P < 0.0001.

To ascertain whether the potent cell migration effects of 80Pz20Bz hydrogels with enhanced network dynamics in vitro can be translated to an accelerated wound-healing response *in vivo*, we administered hydrogels onto full-thickness excisional wounds in diabetic mice (Fig. 5A). Wounds without treatment (Blank) and wounds treated with 0Pz100Bz and 50Pz50Bz hydrogels were used as controls.

We first examinedthe epidermal reconstruction during wound healing, a critical clinical indicator for evaluating wound healing. Digital photographs showed that wounds treated with 80Pz20Bz hydrogels exhibited significantly faster re-epithelialization and wound healing process compared to the other groups (Fig. 5B). By day 5, wound treated with 80Pz20Bz hydrogel initiated re-epithelialization. Wound closure in 50Pz50Bz hydrogel and blank groups occurred simultaneously by day 7, with de novo epithelial crawling observed in both. In contrast, re-epithelialization was delayed in the 0Pz100Bz hydrogel group (Fig. 5C and D). Histological analysis of the regenerated tissue revealed that the epithelial gap in 80Pz20Bz hydrogel-treated wounds on day 7 was significantly smaller than in the other control groups, and completed closure was achieved on day 9 (Fig. 5E and F, Fig. S9A). Next, we focused on the histological comparisons between blank and hydrogel groups. By day 9, the wound gap was almost enclosed by a continuous layer of basal keratinocytes in 80Pz20Bz hydrogel group, whereas keratinocyte migration was significantly slower in the other groups. Wounds treated with 80Pz20Bz hydrogel formed a more mature stratified epithelium composed of keratinocytes that closely resembled healthy epidermis of the normal skin. In the de novo epidermis of the 80Pz20Bz hydrogel group, we clearly observed, from bottom to top, the maturing basement membrane, basal layer, granulosum-spinosum and stratum corneum, which implies the restoration of the structure and function of the epidermal barrier (Fig. 5G). In contrast, epithelial tongue crawling and closure of epithelial gaping were still ongoing in the other control groups. Epidermal thickness is another important indicator for assessing wound healing [39]. During the healing process, de novo epidermis undergoes dynamic changes from thickening to thinning, indicating that the wound is gradually transitioning into the remodeling phase. On day 9, histological staining revealed the thickest epidermis in wounds treated with 80Pz20Bz hydrogel-treated. By day 21, this epidermis had thinned to nearly normal thickness (Fig. S9B and C), which suggests that rapid re-epithelialization facilitates the transition of the wound healing process into the remodeling phase.

The enclosed epidermal barrier can provide both physical protection and signaling molecules for granulation tissue formation in the dermis, directing angiogenesis and cell behavior, and promoting granulation tissue maturation and remodeling [40]. Wound granulation tissue formation was assessed histologically through measurements of granulation tissue thickness. Quantification of granulation thickness by histological staining on day 7 showed that the thickness of de novo granulation tissue was significantly increased in 80Pz20Bz hydrogel group (Fig. S9D). The dermis, the wound space below the epithelium, was filled with granulation tissue on Day 9, suggesting that collagen is being deposited and remodeled. Subsequently, we analyzed cell proliferation and vascularization of the wounds in the blank group, 0Pz100Bz hydrogel and 80Pz20Bz hydrogel groups. As expected, 80Pz20Bz hydrogel induced more Ki67^+^ proliferating cells and CD31^+^ cells than the other groups, suggesting that more neonatal cells and neovascularization occurs to support re-epithelialization and granulation tissue formation in wound healing process (Fig. S10).

Notably, the experimental variables we studied is the varying network dynamics of hydrogels. We found that the re-epithelialization rate of wounds was positively correlated with the hydrogel network dynamics in hydrogel groups (Fig. 5H), which led us to doubt whether wound re-epithelialization was regulated by hydrogel network dynamics. Next, we collected epidermal tissue samples after treating performed transcriptomic sequencing. Enrichment analysis of 80Pz20Bz up-regulated genes (p-value <0.05, ׀Log_2_(fold change)׀ >1) revealed enrichment of skin development, establishment of skin barrier, epidermis development, keratinocyte differentiation and epidermal cell differentiation in the 80Pz20Bz hydrogel group, relative to the blank group and 0Pz100Bz hydrogel group (Fig. 5I).

### 2.5 LZM-PEG hydrogels with enhanced network dynamics accelerates re-epithelialization by promoting epidermal cell migration

**Fig. 6:**
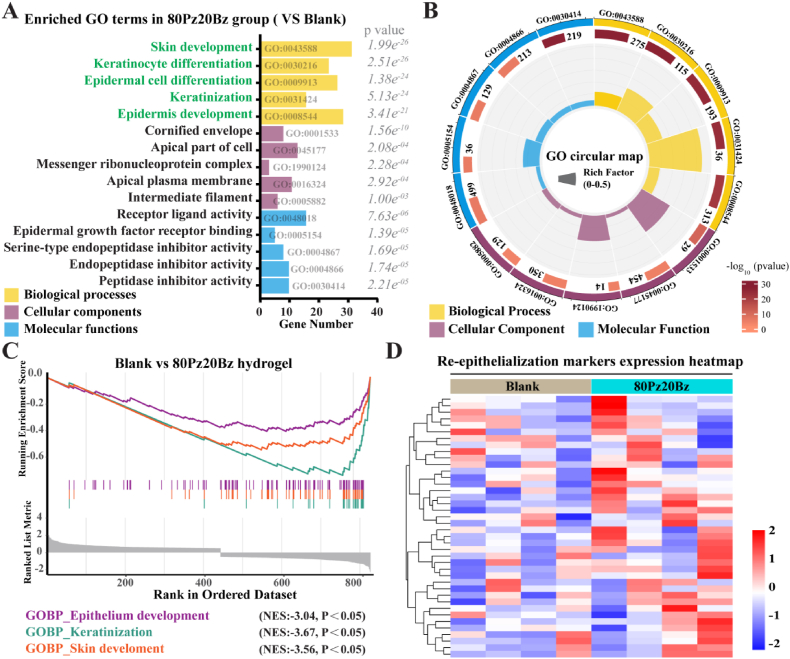
LZM-PEG hydrogels with enhanced network dynamics accelerates re-epithelialization by promoting epidermal cell migration. (A) Enriched 15 GO terms in 80Pz20Bz group. (B) Circular graphics of 15GOterms between group blank and 80Pz20Bz. (C) Gene Set Enrichment Analysis (GSEA) based on RNA-seq data of the blank vs. 80Pz20Bz hydrogel groups, showing significant enrichment of re-epithelialization-related gene sets in the strongly dynamic hydrogel group. (D) Heatmap of the re-epithelializations markers expression in blank and 80Pz20Bz groups. All data are presented as mean ± SD. Statistical significance was calculated by unpaired two-tailed Student's *t*-test. ∗∗∗P < 0.0001.

As we hypothesized, multiple functional terms associated with epidermal repair were significantly enriched in 80Pz20Bz hydrogels group by Gene Ontology enrichment analysis (GO). The top 15 enriched terms compared to the Blank group highlighted genes involved in the regulation of skin wound re-epithelialization across biological processes, molecular functions, and cellular components (Fig. 6A and B). The Gene Set Enrichment Analysis (GSEA) results indicated biological processes associated with epidermal repair and wound re-epithelialization were significantly enriched in 80Pz20Bz hydrogel group compared to blank group (Fig. 6C). We next focus on the differences in hydrogel network dynamics-induced wound re-epithelializationgene expressions, including cell_migration and cell_proliferation. These differential gene expressions are shown in the resulting heatmap (Fig. 6D). Collectively, the histological and bioinformatic analyses based on functional validation suggest that 80Pz20Bz hydrogel accelerates the rapid healing of diabetic skin wounds by activating epithelialization-related functional components and biological processes.

Next, we employed Gene Set Variation Analysis (GSVA) to analyse the signalling pathways activated by 80Pz20Bz hydrogel during its *in vivo* application. Signalling pathways associated with repair and epithelialization, including *gap junction*, *EMT down*, *adherens junction*, and *apical junction*, were upregulated (Fig. S11A) [41]. In addition, we conducted a chronological analysis of epithelialization-related signalling pathway activation using Single Sample Gene Set Enrichment Analysis (ssGSEA). The 80Pz20Bz hydrogel group exhibited significantly higher scores on day 3 compared to the blank group (Fig. S11B), suggesting that the contribution of 80Pz20Bz hydrogel to wound re-epithelialization was predominantly observed between days 3 and 7. Consistent with this finding, de novo epidermis formation was evident in wounds treated with 80Pz20Bz hydrogel by day 5.

**Fig. 7:**
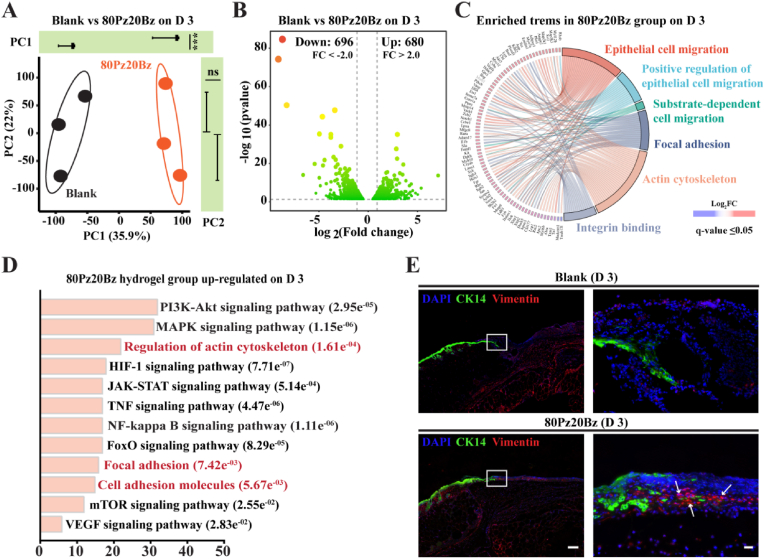
LZM-PEG hydrogels with enhanced network dynamics accelerates re-epithelialization by promoting epidermal cell migration. (A) PCA plot of RNA-sequencing samples from blank and 80Pz20Bz hydrogel group on day 3 (n = 3). (B) Gradient volcano plot of RNA-sequencing samples from blank and 80Pz20Bz hydrogel group on day 3. (C) GO terms enriched in 80Pz20Bz hydrogel group. (D) KEGG signaling pathways enriched in 80Pz20Bz hydrogel group. (E) Representative fluorescent images of CK14 and Vimentin from the different groups on day 3. (Left: Scale bar: 200 μm; Right: Scale bar: 20 μm). All data are presented as mean ± SD. Statistical significance was calculated by unpaired two-tailed Student's *t*-test. ∗∗∗P < 0.0001.

Given that re-epithelialization occurs at an early stage of the wound healing process, to further elucidate the biological mechanisms by which hydrogels promote epithelialization during the initial phases of wound healing, we conducted an in-depth analysis of sequencing data obtained from tissue samples collected on day 3. The results of Principal Component Analysis (PCA) demonstrated a clear distinction between the 80Pz20Bz hydrogel and the Blank samples along the two canonical axes that optimally differentiate the groups (Fig. 7A). We then performed further analysis and found that there were 696 down-regulated and 680 up-regulated differentially expressed genes (DEGs) in the comparison between the blank and 80Pz20Bz hydrogel groups (׀Log_2_(fold change)׀≥2) (Fig. 7B). GO enrichment analysis indicated significant enrichment of terms related to cell migration, including epithelial cell migration, positive regulation of epithelial cell migration, and focal adhesion (Fig. 7C). Additionally, Kyoto Encyclopedia of Genes and Genomes (KEGG) pathway analysis identified upregulation in signaling pathways associated with actin cytoskeleton regulation, focal adhesion, and cell adhesion molecules (Fig. 7D), which are closely linked to epithelial cell migration. The immunofluorescence staining results for vimentin and keratin 14 demonstrated that the wounds treated with 80Pz20Bz hydrogel exhibited a higher number of positively stained epithelial cells on day 3. This suggests that the 80Pz20Bz hydrogel can effectively facilitate the recruitment and migration of epithelial stem cells to the injured area *in vivo* (Fig. 7E). Together, these results provide further evidence that faster hydrogel network dynamicscan more effectively facilitate the migration of epithelial cells at the wound edge through mechanical forces, thereby accelerating the re-epithelialization process.

## 3 Discussion

Our study demonstrates that LZM-PEG hydrogels with enhanced network dynamics effectively overcome the pathological mechanical barriers in diabetic wounds by mimicking dynamic ECM-cell mechanical interactions, thereby accelerating re-epithelialization through mechanotransduction activation. Our findings corroborate emerging evidence that viscoelastic matrix properties regulate cellular traction force generation and cytoskeletal remodeling, while extending current knowledge by establishing a direct correlation between hydrogel network dynamics and the spatiotemporal progression of epidermal migration in chronic wounds. Specifically, cell adhesion mediated by FAK activation and subsequent cell contraction promotes integrin clustering, initiating downstream biological events [42]. Stress-relaxed hydrogel networks enhance nascent protein secretion by activating cellular mechanotransduction promptly, leading to morphological changes and migration. This mechanism circumvents the need for exogenous force application-a critical advancement over existing mechanotherapy strategies-by leveraging intrinsic material dynamics to sustain mechanochemical signaling loops between cells and matrices.

The dynamic mechanical properties of hydrogels crosslinked by reversible bonds are crucial for regulating cell behaviors. Our hydrazone-based hydrogels with tunable stress relaxation enabled us to probe how matrix dynamics affect cellular responses. Yang et al. [27] demonstrated through combined molecular dynamics and kinetic Monte Carlo simulations that kinetic binding constants (*k*_*on*_*/k*_*off*_) rather than equilibrium binding constant—govern network reorganization and enable rapid cellular protrusion by facilitating force-dependent “gate-opening” events. Our system demonstrates that faster stress relaxation promotes cell migration. This aligns with the idea that rapid crosslink dissociation facilitates matrix remodeling and adhesion maturation. These findings underscore the importance of dissociation kinetics in designing dynamic hydrogels for cell-instructive applications. This consistency underscores the central role of dissociation kinetics in guiding matrix remodeling and adhesion maturation, supporting the design of kinetic-controlled hydrogels for predictive cell mechanobiology.

The interface interaction of encapsulated cells with engineered hydrogels is known to be relevant in explaining the observed phenomena [35,43,44]. The observed upregulation of nascent adhesion proteins at hydrogel interfaces indicates that stress relaxation promotes focal adhesion turnover, a critical step in cell migration through stiffened diabetic tissues. This effect may synergize with the time-dependent mechanical unloading by the hydrogel to reduce actomyosin contractility, thereby enabling persistent directional migration despite microenvironmental MMP/TIMP imbalances. Importantly, our *in vivo* data demonstrate that early-stage modulation of cytoskeletal reorganization precedes complete re-epithelialization, suggesting that hydrogel-induced mechanical priming initiates a self-sustaining repair cascade. The rapidly stress-relaxed LZM-PEG hydrogel provides more anchor points for epithelial cells at the wound margins to ‘grab’ and ‘pull’, leading to cell 3D infiltration and/or surface crawling towards the hydrogel. Such temporal coordination between material dynamics and biological responses highlights the importance of designing biomaterials with viscoelastic profiles tailored to specific disease stages.

Hydrogel pore size is critical for cell migration and nutrient diffusion, with large pores (> tens of micrometers) generally required for efficient infiltration [45]. By controlling precursor concentration and crosslinking, we matched initial modulus and pore size across groups (Fig. S12) to isolate stress relaxation behavior (τ_1_/_2_) as the key variable. We observed that under conditions of equivalent initial pore size, hydrogels with faster stress relaxation rates (80Pz20Bz group) still markedly enhanced cell migration and epithelial regeneration. This finding suggests that, beyond basic architectural requirements, dynamic mechanical properties of the hydrogel network dominate cellular functions. Future studies should probe the synergy between pore size and stress relaxation kinetics—specifically, whether refining pore dimensions within an optimized kinetic regime can potentiate tissue regeneration.

Chronic inflammation represents a fundamental pathological feature in diabetic skin wounds [46,47]. Given the importance of modulating the inflammatory microenvironment during wound healing, we examined whether LZM-PEG hydrogels could regulate macrophage polarization. Although intergroup differences in M2/M1 macrophage biomarkers were observed in vitro, the early promotion of M2 polarization by the 80Pz20Bz hydrogel at 12 h was downregulated by 24 h. None of the hydrogels promoted M1 polarization (Fig. S13A). ssGSEA analysis of wound macrophages, M1/M2 polarization and inflammation-promoting revealed no significant changes in infiltration or polarization of macrophages at days 3 and 7 (Fig. S13B). These results suggest that LZM-PEG hydrogels accelerate wound healing by modulating stromal cell behaviour, which contrasts with previous paradigms that emphasised M2 macrophage polarization.

## 4 Conclusions

In summary, we presented in this study a novel LZM-PEG hydrogel platform to offeraninnovative approach to enhance epithelial cell migration and accelerate the healing of chronic wounds by harnessing the viscoelastic properties of these hydrogels to modulate the mechanical environment at the wound site. This strategy holds the potential to establish a new paradigm for tissue repair, particularly in the complex context of diabetic wound healing. However, further research is essential to refine these materials and thoroughly investigate their clinical applicability, with key areas of focus including the long-term effects of these hydrogels on wound healing, especially concerning potential immune responses and chronic inflammation. Additionally, the mechanisms by which ECM network dynamics influence other cellular behaviors, such as proliferation and differentiation, remain inadequately understood and merit more detailed investigation. Despite these considerations, the results obtained from this research provide a strong foundation for the development of next-generation active materials aimed at enhancing tissue regeneration and recovery, paving the way for innovative therapeutic strategies that address the pressing challenges associated with chronic wounds.

## 5 Experimental section

*Hydrogel preparation:* For LZM -PEG hydrogel, pre-purifiedLysozyme (14,307 Da) was co-incubated at 37°Cwith SC-PEG-P or SC-PEG-B (2 kDa) in DMEM (Gibcao) to obtain precursors 1(LZM-P) and 1(LZM-B). Terminal-modified hydrazide eight-armedPEG (20 kDa)and 4-amino-DL-phenylalanine were solubilized in phosphate buffered saline buffer as precursor 2. All precursors were mixed according to predetermined formulations yielding different stress-relaxing hydrogels. All hydrogels possessed a solid content of 0.1 wt/vol, ensuring consistent initial elastic modulus.

*Characterization of hydrogels:* The G′ and G″ of hydrogels were monitored at a fixed strain of 1 % and frequency of 1 Hz. Stress relaxation measurements were performed by time sweep tests at a constant initial strain of 10 % and a fixed frequency of 1 Hz. The corresponding stress relaxation curves were then normalized to the initial value.

To simulate the swelling behavior of hydrogels in the wound, hydrogels of each formulation with the same volume were loaded in homemade molds. All hydrogels were incubated with DMEM at 37 °C. The initial weight of hydrogels was noted as W_0_ and the weight of the hydrogel at each timepoint was noted as W_1_. The residual mass of hydrogels was calculated as follows.

$$\text{Swelling}\;\text{ratio}\;\left(\%\right)=W_{1}/W_{0}\times 100\%.\;\left(n=3\right)$$

To investigate the nutrient diffusion, all hydrogels were immersed in the Rhodamine B (1.0 mg L^−1^ in PBS; Rhawn) at 37 °C, then taken out at each timepoint. 1 mL Protease K (0.1 %) was added for hydrogel degradation. After the hydrogels were completely degraded, the absorbance of each sample was measured at a wavelength of 554 nm. 0Pz100Bz hydrogels in Protease K solution were set as the controls.

*Fluorescent bead tracking:* For visualizing the hydrogel network dynamics, 1 μmfluorescent beads (MCE, Cat^#^L9654) were homogeneously mixed into the cell-free hydrogel precursors at a same concentration before it was loaded in the holes (d = 21 mm) of the 12-well plate (Fig. S14). A cylindrical blank area (d = 2 mm) was then excised from hydrogel center using a homemade mold and refilled with cell-loaded hydrogel. Bead displacements were then tracked using microscope. Relative deformations at time t = 1 day were calculated with respect to the initial blank area at time t = 0.

*Live/Dead assay of encapsulated cells:* The cell density of cell-laden hydrogelwas 3 million cells/mL. Hydrogel constructs werecultured in DMEM supplemented with 10 % FBS and 1 % penicillin/streptomycin. According to the manufacturer's protocol, we used Calcein-AM/PI staining kit (Beyotime) and lactate dehydrogenase (LDH) cytotoxicity test kit (Servicebio) to assess cell viability. Fluorescence images were acquired with aconfocal microscope and analyzed using ImageJ.

*2D spreading assay:* To investigate the effects of LZM-PEG and BSA-PEG hydrogel on cell adhesion, cell clusters, centrifuged and not thoroughly dispersed, were seeded onto hydrogel. To assess the effects of 80Pz20Bz and 0Pz100Bz LZM-PEG hydrogels on cell spreading, single cells, centrifuged and thoroughly dispersed, were seeded onto hydrogel.

*Interface migration assay:* HaCat or L929 cell spheroids were loaded to the bottom of the Petri dish substrate before molding. After the polymerization of spheroids was completed, the hydrogel was injected to cover the spheroids and was incubated. Cell migration from spheroids was monitored by electron microscope during culture.

*Wound healing assay:* Cells were seeded and cultured ahead of time. Once the cell monolayer nearly formed, cells were starved in a serum-free medium for 12 h. Subsequently, scratches were created using the tip of a 200 μL pipette, floating cells were washed away, then hydrogel was injected into the scratches. Cells were finally incubated in serum-free DMEM. Scratch closure was monitored with a microscope.

*3D interface traversal assay:* A homemade platinum sheet was used to separate the well slots at the center. Matrigel was mixed with cells injected on the left side of the platinum sheet and incubated in the incubator at 37 °C to allow gel formation. After 24 h of incubation, the platinum sheet was removed and cell-free hydrogel was deposited on the right side. Once gelation was complete, the medium was added for incubation. Cell traversal was recorded at set time points by microscope.

*3D migration assay:* (1) HaCat and/or L929 cell spheroids were encapsulated into hydrogel before polymerization. Hydrogel constructs were cultured at 37 °C/5 % CO_2_. Cell migration was recorded at set time points by microscope. For inhibition studies, Blocking of cytoskeletal contraction was achieved using 80 μM Blebbistain (MCE, HY-13441), which was replenished daily. (2) HaCat cells were adhered to the surface of the dextran spheres. Subsequently, dextran spheres were encapsulated into hydrogel before hydrogel polymerization. Hydrogel constructs were cultured at 37 °C/5 % CO_2_. Cell migration was recorded at set time points by electron microscope.

*Nascent protein labeling and antibody inhibition:* For nascentproteinlabeling, l-azidohomoalanine (AHA, Beyotime) was added during cell culture. 30 μM DBCO-488 (Click Chemistry Tools, MCE, HY-D2171A) was then added into cell-laden hydrogels followed by 40 min incubation at 37 °C/5 % CO_2_. Hydrogels were fixed with 4 % paraformaldehyde for 30 min at room temperature after washing in PBST. Following three washes with PBST, fluorescent images were acquired with aconfocal microscope. Protein transport and secretion were blocked using 120 nM 2-(4-fluorobenzoylamino)-benzoic acid methyl ester (Exo-1, MCE, HY-112670), which was replenished daily.

*Immunofluorescence for fixed cells:* For immunofluorescence analysis, cell samples were fixed with 4 % paraformaldehyde for 15 min at room temperature (3D cultured cells were fixed for 30 min) and washed with PBST three times. Subsequently, cell samples were permeabilized with a permeabilizing solution (Beyotime) for 15 min and washed three times with PBST (3D cultured cells were permeabilized for 30 min). 5 % (w/v) Goat serum in PBSTwas then added to minimize non-specific staining, after which primary antibody anti-FAK(Cell signaling technology), anti-pFAK (Cell signaling technology), and anti-Paxillin (MedChemExpress), anti-Integrinβ1 (Cell signaling technology) in PBST were added to the constructs and incubated at 4 °C overnight. Cell samples were then washed three times with PBST. Secondary antibody goat anti-rabbit AlexaFluor® 555 (Abcam), FITC-phalloidin (Biosharp) and DAPI (Biosharp) were added and incubated at room temperature. Cell samples finally were washed with PBST. Fluorescent images were acquired with a confocal microscope and analyzed using ImageJ.

*RNA isolation and quantitative reverse transcription PCR:* Total RNA was isolated using RNAiso Plus (TaKaRa) according to the manufacturer's instructions. cDNA was synthesized using the iScript1™ cDNA Synthesis kit (Bio-Rad). Relative gene expression was determined using the iTaq Universal SYBRGreen Supermix (Bio-Rad). Gene amplification and quantification of cDNA was performed on a CFX96 touch PCR detection system. The relative expression level for each gene (fold change) was calculated using the comparative 2^−△△^ method. All experiments were conducted in triplicate to obtain the average data. The gene-specific primers used are listed in Table S1.

*Chronic wound-healing of diabetic mice:* The experimental protocol was approved by the Shanghai Changhai Hospital Ethics Committee (Approval No. CHEC(A.E)2023-020). Eight-week-old male db/db (C57BL/6) mice were purchased from Shulaibao (Wuhan) Biotechnology and used in the study. Following anesthesia and hair removal, the mice were constructed with an 8-mm-diameter full-layer skin defect wound on their backs. Pre-determined target hydrogels were injected directly into the defect area. No implants were placed in the blank group. Photographs of the wounds were taken at regular intervals, and the wound healing rate was calculated using the formula: wound healing rate (%)=(1- area of unhealed wounds/original area)x 100 %. Mice were euthanized at 3, 7, 9, and 21 days (24 days for 0Pz100Bz hydrogel group) for tissue sampling. Circular skin samples containing all skin layers were removed and centered on the residual wound for histological assessment and subsequent RNA sequencing (RNA-seq).

*Statistical analysis:* The statistical significance of data was analyzed using analysis of Ordinary one-way ANOVA and two-tailed Student's t-test at the 95 % confidence level using GraphPad Prism 9.0 (GraphPad Software, San Diego, CA, USA), with significant thresholds set at ∗P < 0.05,∗∗P < 0.01, ∗∗∗P < 0.001, and ∗∗∗∗P < 0.0001.

## CRediT authorship contribution statement

**Songsong Shi:** Writing – review & editing, Writing – original draft, Visualization, Investigation, Formal analysis, Data curation. **Wei Zhang:** Investigation, Data curation. **Yuanman Yu:** Writing – review & editing, Methodology, Investigation. **Jiaqi Qiu:** Formal analysis. **Runzhi Huang:** Data curation. **Shizhao Ji:** Resources, Project administration, Conceptualization. **Xue Qu:** Writing – review & editing, Supervision, Resources, Methodology, Conceptualization.

## Ethics approval and consent to participate

All animal experiments were conducted in accordance with the NIH guidelines for the care and use of laboratory animals (NIH Publication no. 85-23 Rev. 1985). The experimental protocol was reviewed and approved by the Animal Ethics Committee of Shanghai Changhai Hospital, affiliated with Naval Medical University, Shanghai, China (Approval No. CHEC(A.E)2023-020).

## Declaration of competing interest

Authors declare no conflict of interest.
