# Supplementary material for: Dynamic matrix engineering promotes nascent protein deposition to drive cell migration and expedite Re-epithelization in chronic wound
Source: Bioact Mater. 2025 Oct 28;56:455–67. doi: 10.1016/j.bioactmat.2025.10.020 (PMC12597304; doi:10.1016/j.bioactmat.2025.10.020)
Supplement: Multimedia component 1 [file mmc1.docx]

Supporting Information for

**Dynamic Matrix Engineering Promotes Nascent Protein Deposition to Drive Cell Migration and Expedite Re-Epithelization in Chronic Wound**

**This PDF file includes:**

Figures S1 to S11

Tables S1


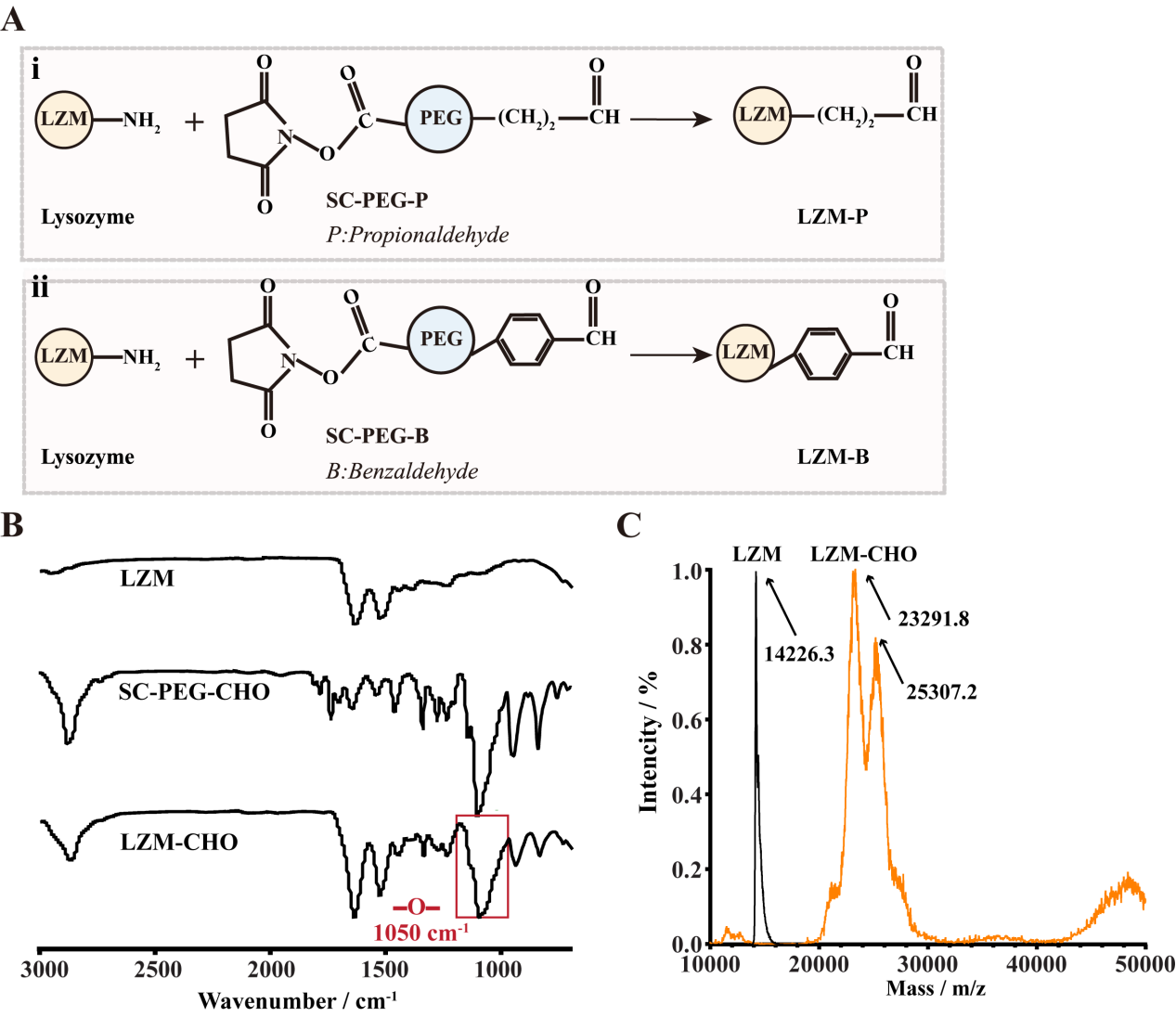


**Figure S1. A)** Modification of LZM via amidation with SC-PEG-P or SC-PEG-B to form LZM-P and LZM-B, respectively. **B)** FT-IR spectra demonstrate the successful aldehyde modification of LZM. Specifically, a prominent absorption band at approximately 1050 cm^-1^ corresponding to the ether bond in SC-PEG-B is observed in LZM-B, confirming the modification. **C)** Mass spectrometric analysis shows a significant increase in molecular weight of LZM-CHO, with peaks at 23291.8 Da and 25307.2 Da, indicating the successful conjugation of aldehyde groups to the lysozyme.

**
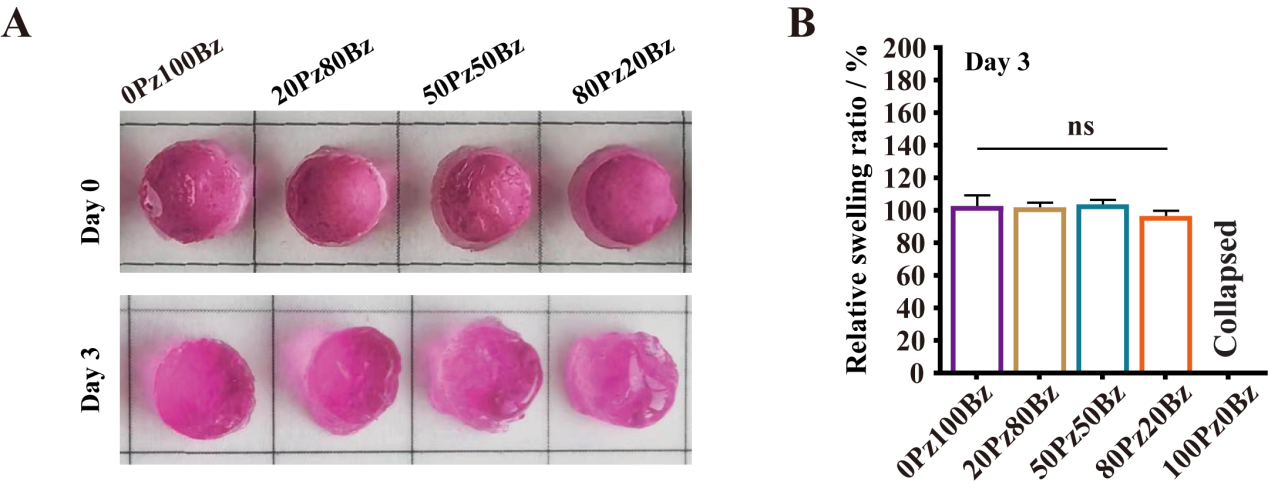
**

**Figure S2. A)** Representative photographs of LZM-PEG hydrogels showing their swelling behavior over 3 days. **B)** Swelling ratio measurements indicate no significant differences in hydrogel swelling after 3 days across different formulations, except for the 100Pz0Bz hydrogel, which undergoes disintegration. (n=3) Statistical significance is set as ^*^p < 0.05, ^**^p < 0.01, ^***^p < 0.001 and ^****^P < 0.0001; ns, not statistically significant.

**
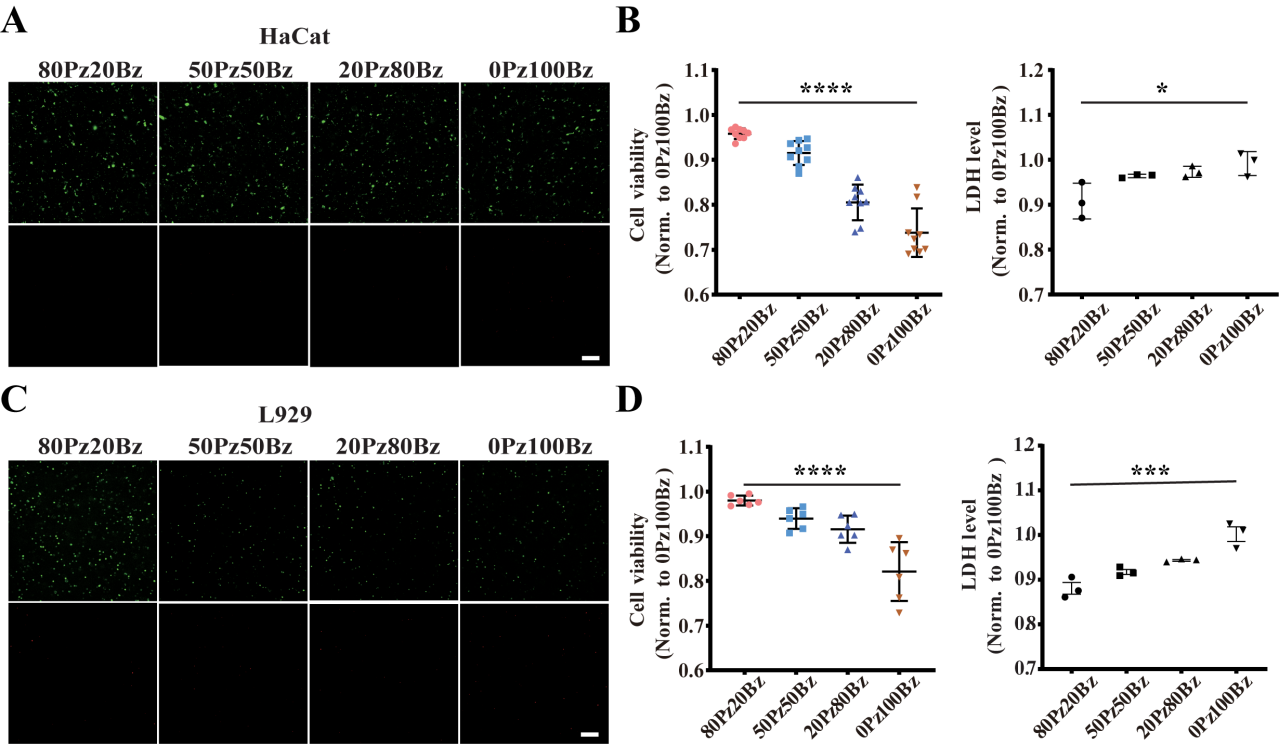
**

**Figure S3. A)** Representative Live-dead staining of keratinocytes encapsulated in LZM-PEG hydrogels on culture day 1. Live: green; dead: red. Scale bar, 100 μm. **B)** Representative Live-dead staining of fibroblasts encapsulated in LZM-PEG hydrogels on culture day 1. Live: green; dead: red. Scale bar, 100 μm. **C)** Left: quantification of the viability of encapsulated cells calculated as the percentage of live keratinocytes among all stained cells (n=9); Right: quantification of LDH levels on culture day 1, normalized by the value in the slowest relaxing gel condition. (n=3) **D)** Left: quantification of the viability of encapsulated cells calculated as the percentage of live fibroblasts among all stained cells (n=6); Right: quantification of LDH levels on culture day 1, normalized by the value in the slowest relaxing gel condition (n=3). These evidencess suggest that both cells have better proliferative viability in the highly dynamic LZM-PEG hydrogels. All data are presented as mean ± SDs. Statistical significance is set as ^*^p < 0.05, ^**^p < 0.01, ^***^p < 0.001 and ^****^P < 0.0001; ns, not statistically significant.

**
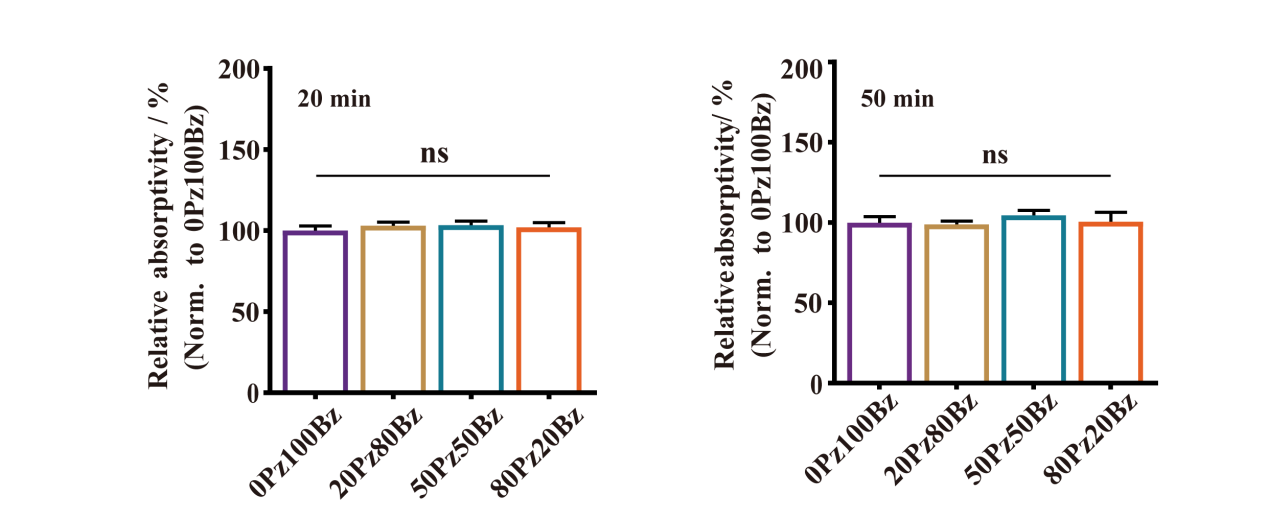
**

**Figure S4.** The diffusion rates test suggests that nutrient diffusion is consistent across hydrogels with varying network dynamics. (n=3) All data are presented as mean ± SDs. Statistical significance is set as ^*^p < 0.05, ^**^p < 0.01, ^***^p < 0.001 and ^****^P < 0.0001; ns, not statistically significant.

**
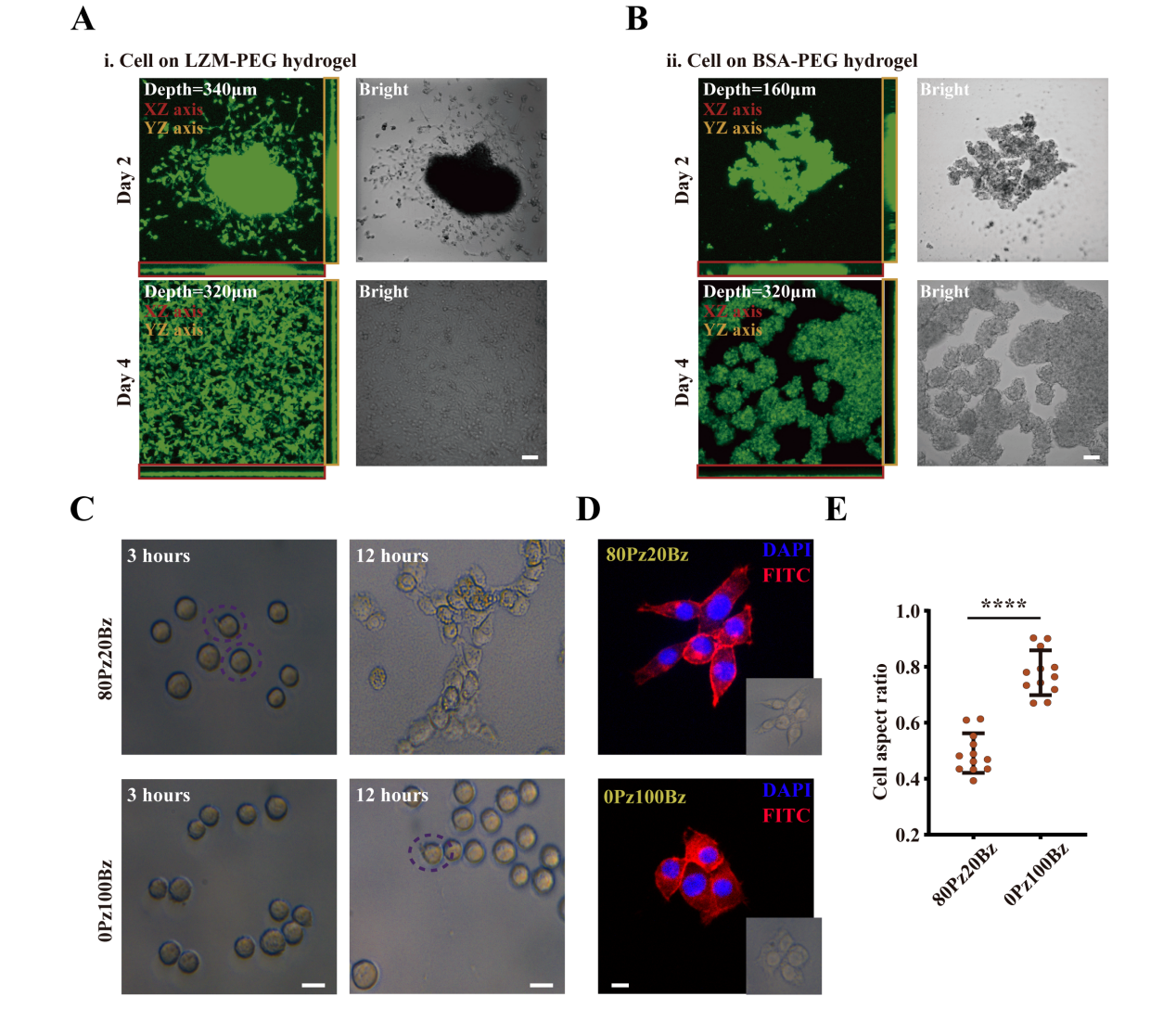
**

**Figure S5. A)** Representative images of L929 cell clusters spreading and infiltration on LZM-PEG hydrogels on culture days 2 and 4. Green:F-actin. Left:immunofluorescent labeling and 3D reconstruction; Right: Bright. Scale bar, 100 μm. **B)** Representative images of L929 cell clusters spreading and infiltration on BSA-PEG hydrogels (which were prepared by using the similar protocals illustrated in Figure **1A** and Figure **S1**) on culture days 2 and 4. Green:F-actin. Left:immunofluorescent labeling and 3D reconstruction; Right: Bright. Scale bar, 100 μm. **C)** Representative images of L929 cell spreading on LZM-PEG hydrogels at 3 and 12 hours of incubation. Scale bar, 25 μm. **D)** Representative images of L929 cell spreading on LZM-PEG hydrogels at days 2 of incubation. Red: F-actin; Blue: Nuclei. Scale bar, 10 μm. **E)** The aspect ratio of L929 cells on LZM-PEG hydrogels (n=12). These evidencess suggest that LZM-PEG hydrogels with enhanced network dynamics promote cell adhesion and spreading. All data are presented as mean ± SDs. Statistical significance is set as ^*^p < 0.05, ^**^p < 0.01, ^***^p < 0.001 and ^****^P < 0.0001; ns, not statistically significant.

**
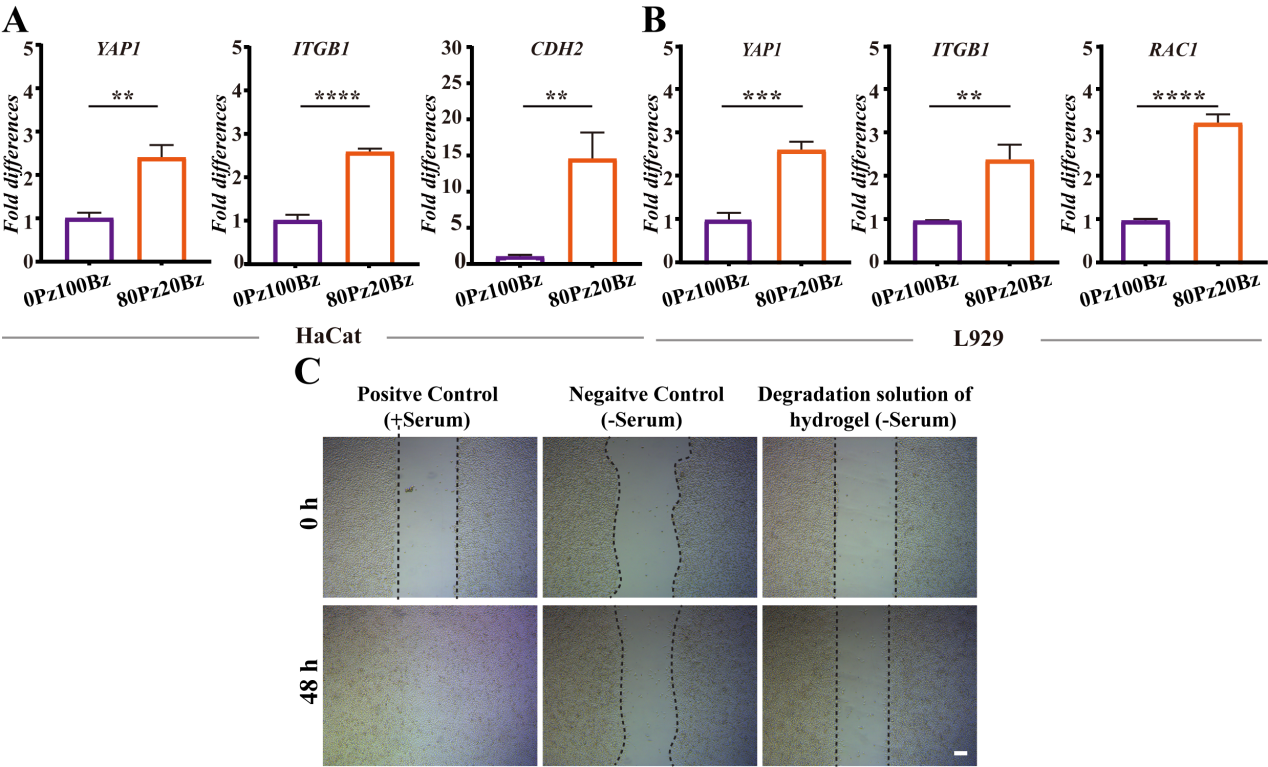
**

**Figure S6. A)** qRT-PCR analysis of *Yap1*, *ITGβ1*, and *N-cadherin* of HaCat cell in model **i** from the different groups. (n=3) **B)** qRT-PCR analysis of *Yap1*, *ITGβ1*, and *Rac1* of L929 cell in model **i** from the different groups (n=3). Evidence from **A)** and **B)** for up-regulation of cell migration-related genes in response to highly dynamic hydrogels. **C)** Representative images of L929 cell migration in model **i** from the different groups suggest that cell migration is caused by cell-hydrogel direct interactions rather than hydrogel degradation products. Scale bars, 100 μm. All data are presented as mean ± SDs. Statistical significance is set as ^*^p < 0.05, ^**^p < 0.01, ^***^p < 0.001 and ^****^P < 0.0001; ns, not statistically significant.


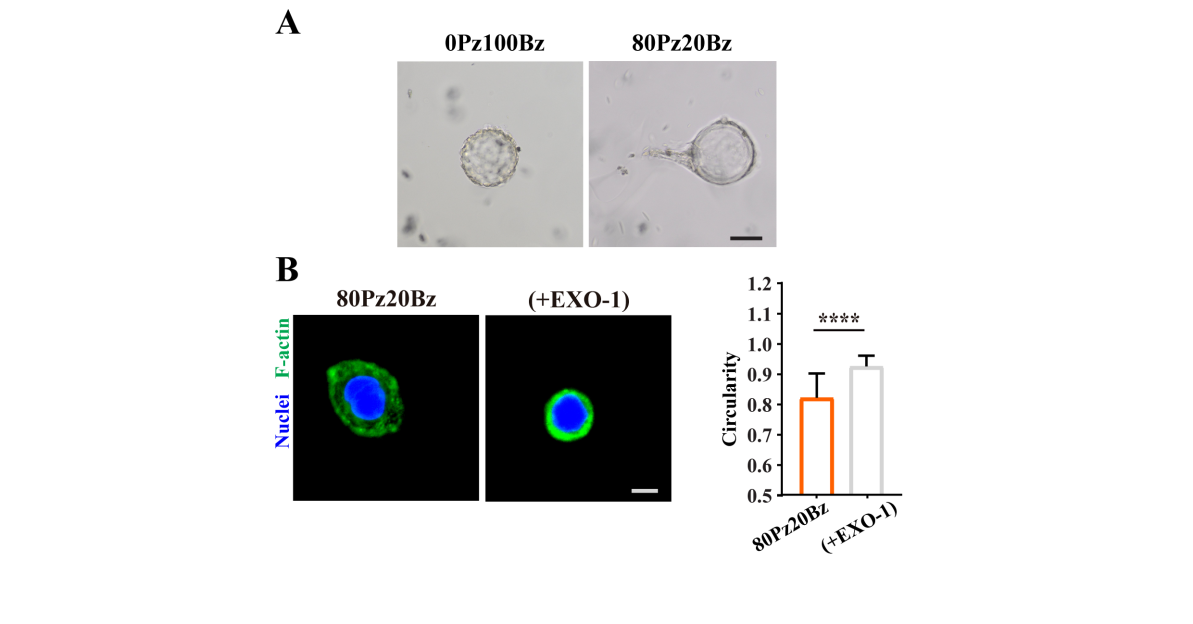


**Figure S7. A)** Representative bright-field images of HaCat cell migration. **B)** Representative fluorescent images and quantification of HaCat cell suggest that EXO-1 treatment inhibits cytoskeletal remodeling. Scale bar, 10 μm. (n=3) All data are presented as mean ± SDs. Statistical significance is set as ^*^p < 0.05, ^**^p < 0.01, ^***^p < 0.001 and ^****^P < 0.0001; ns, not statistically significant.


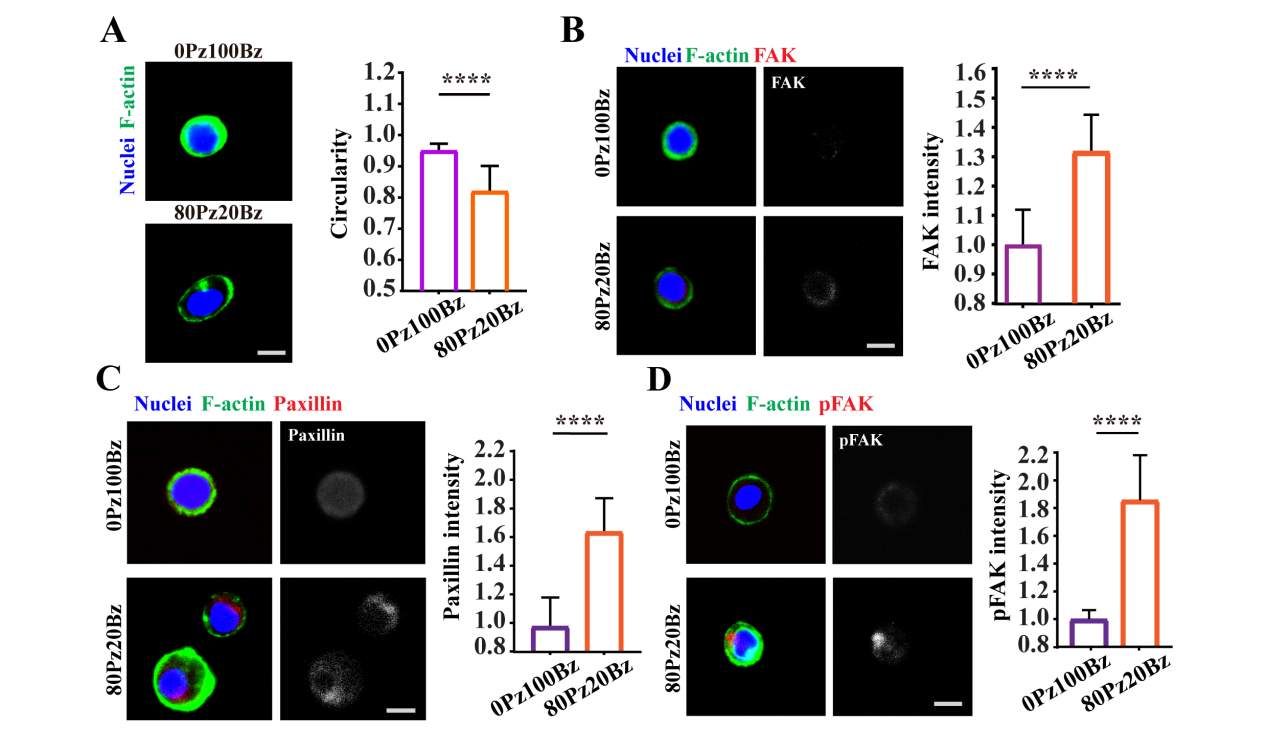


**Figure S8. A)** Representative fluorescent images and quantification of cytoskeleton show that cells in strongly dynamic hydrogels exhibit more pronounced cytoskeletal remodeling. Scale bar, 10 μm. (n=18) **B)** Representative fluorescent images and quantification of FAK in early HaCat cell cultures. Scale bar, 10 μm. (n=8) **C)** Representative fluorescent images and quantification of paxillin in early HaCat cell cultures. Scale bar, 10 μm. (n=8) **D)** Representative fluorescent images and quantification of pFAK in early HaCat cell cultures. Scale bar, 10 μm. (n=8) All data are presented as mean ± SDs. Statistical significance is set as ^*^p < 0.05, ^**^p < 0.01, ^***^p < 0.001 and ^****^P < 0.0001; ns, not statistically significant.

**
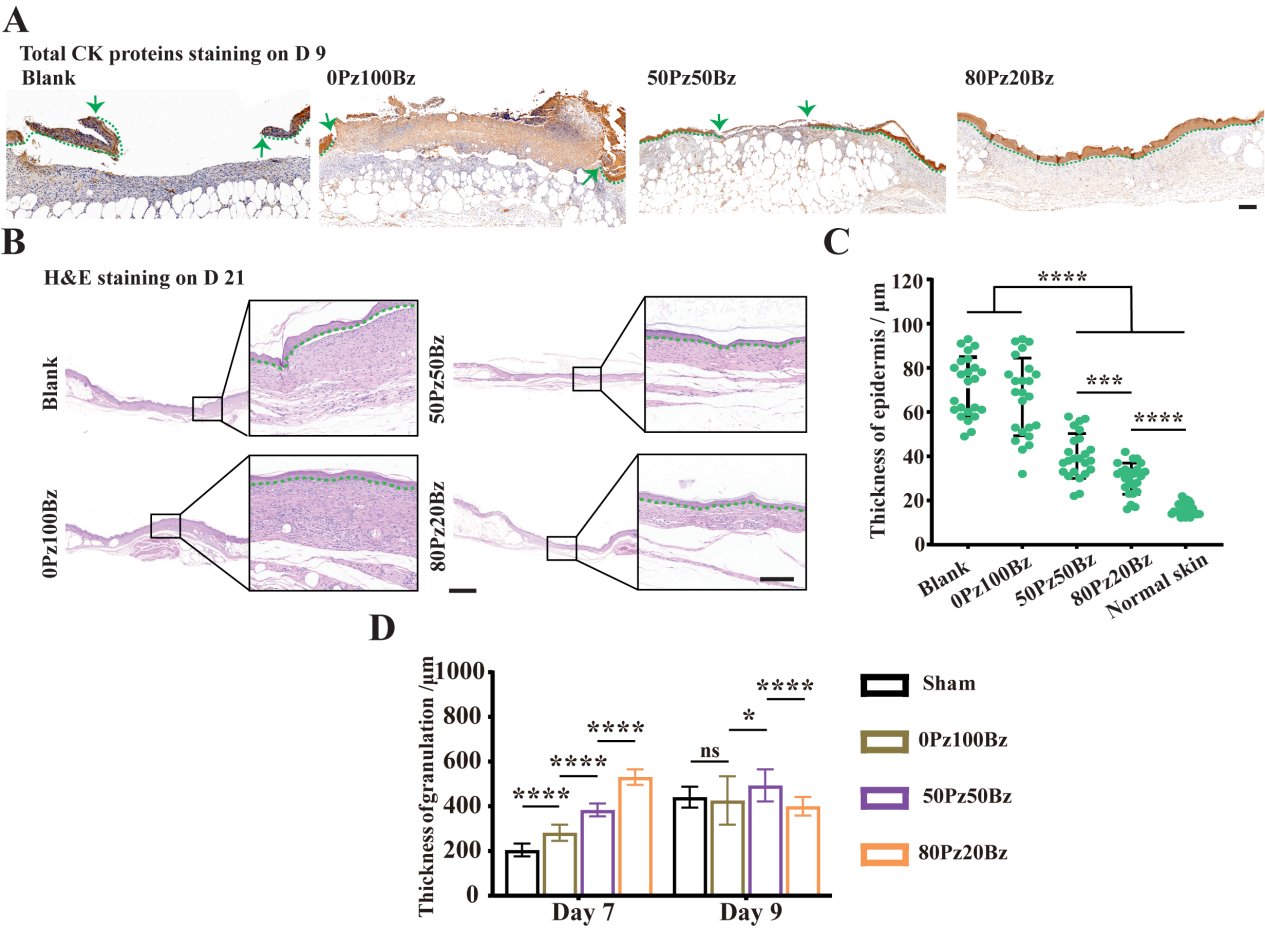
**

**Figure S9.**  **A)** Representative images of total CK peoteins staining from different groups at days 9. Scale bar, 200 μm. **B)** Representative H&E images from different groups at days 21. Left: Scale bar, 1 mm; Right: Scale bar, 200 μm. **C)** Corresponding analysis of regenerated epidermal thickness from different groups at days 21 show that the thickness of the regenerated epidermis in the wound of the strong dynamic hydrogel group is closer to the normal skin epidermis. **D)** Quantification of wound granulation thickness at day 7 and day 9. All data are presented as mean ± SDs. Statistical significance is set as ^*^p < 0.05, ^**^p < 0.01, ^***^p < 0.001 and ^****^P < 0.0001; ns, not statistically significant.

**
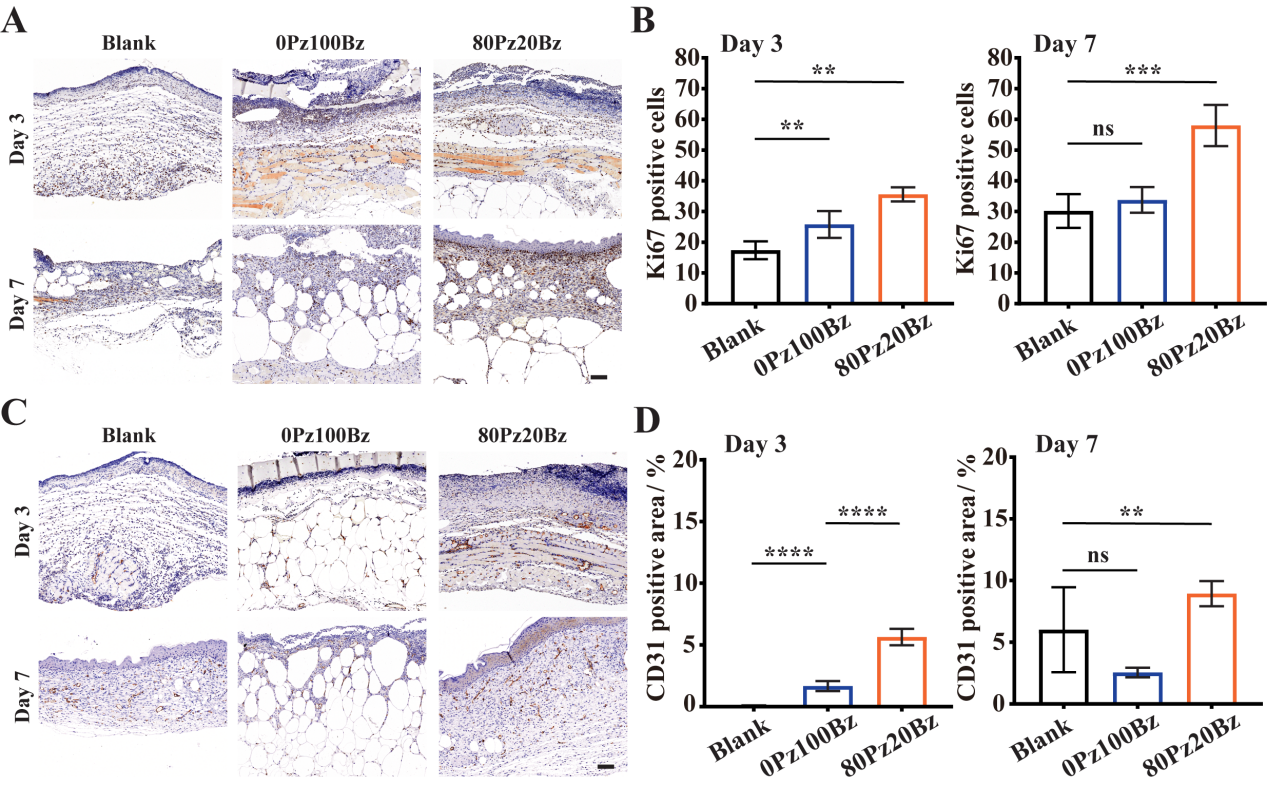
**

**Figure S10. A)** Representative images of Ki67 staining from different groups at days 3, 7. **B)** Correspondence analysis of Ki67^+^ cells from different groups at days 3, 7. (n=5) **C)** Representative images of CD31 staining from different groups at days 3, 7. **D)** Correspondence analysis of CD31^+^ area from different groups at days 3, 7. (n=5). All data are presented as mean ± SDs. Statistical significance is set as ^*^p < 0.05, ^**^p < 0.01, ^***^p < 0.001 and ^****^P < 0.0001; ns, not statistically significant.

**
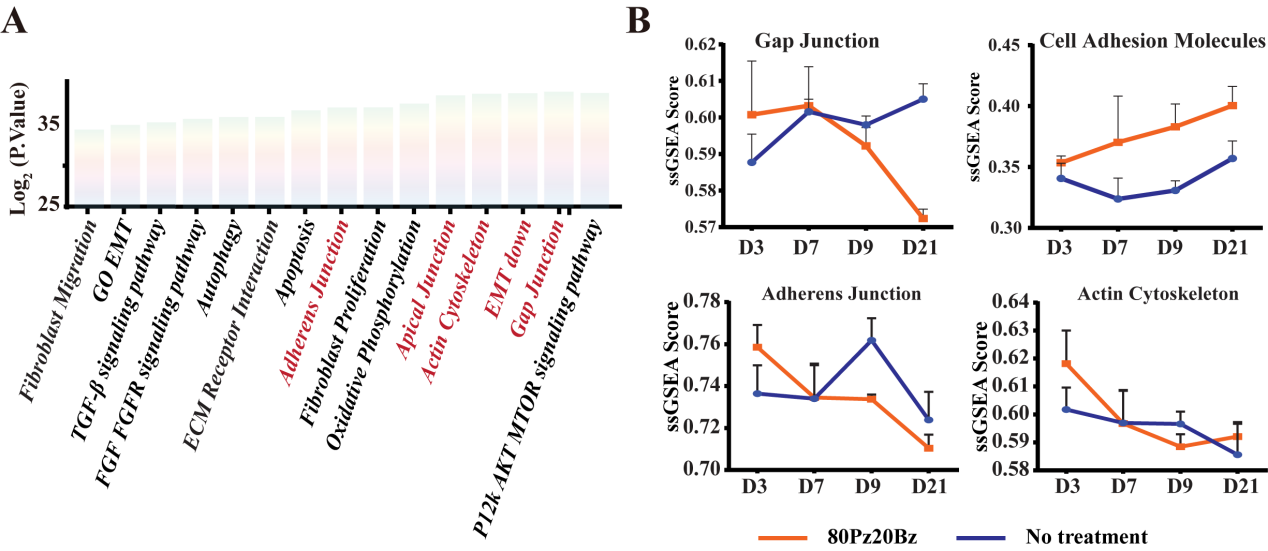
**

**Figure S11. A)** GSVA analysis in 80Pz20Bz hydrogel group suggest that signaling pathways including cell adhesion, cytoskeletal changes are activated. **B)** Corresponding analysis of ssGSEA scores on days 3,7,9 and 21 indicate that gap junction, cell adhesion molecules, adherens junction and actin cytoskeleton are activated. (n=3) All data are presented as mean ± SD.


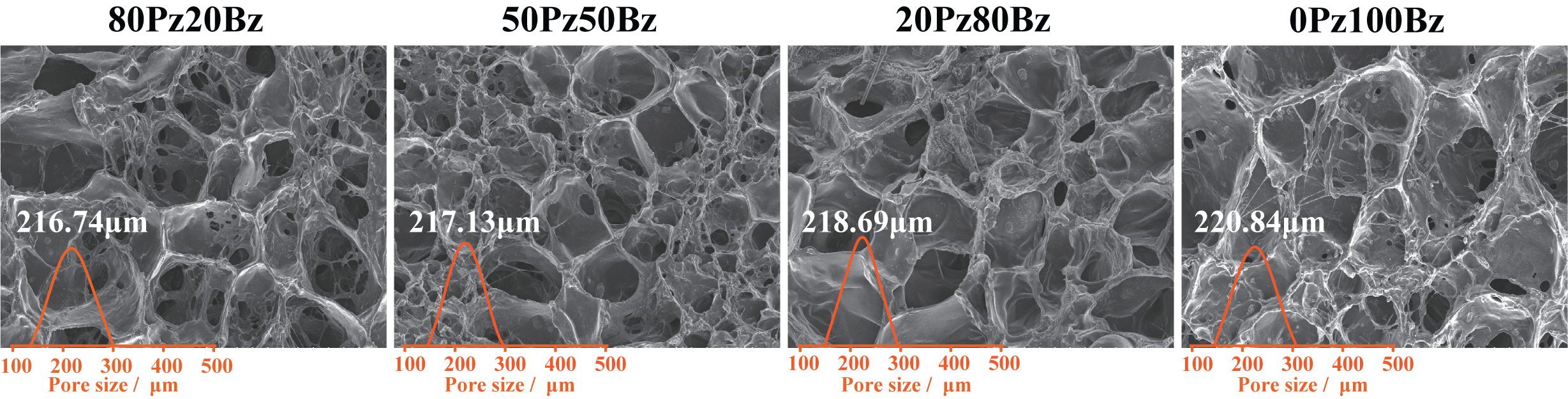


**Figure S12.**  Images captured by SEM show that the pore sizes of the hydrogels in each group are consistent.


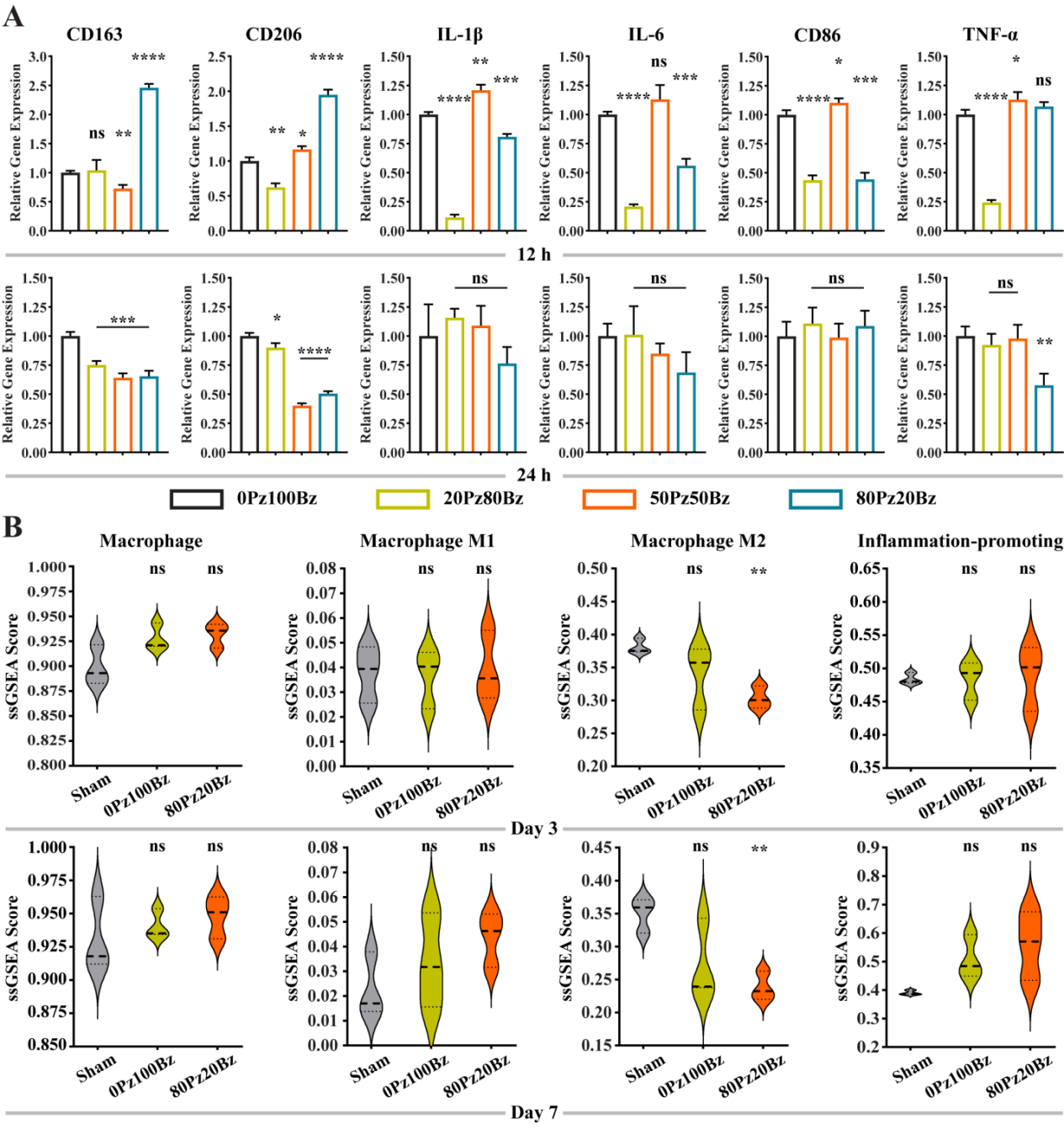


**Figure S13. A)** QRT-PCR analysis of RAW246.7 encapsulated in hydrogel. (Biomarkers of macrophage M2 polarization: CD163, CD206; Biomarkers of macrophage M1 polarization: IL-1β, IL-6, CD86 and TNF-α;) n=3 **B)** The ssGSEA scores for macrophages, M1 macrophages, M2 macrophages, and inflammation-promoting across all wound groups. n=3


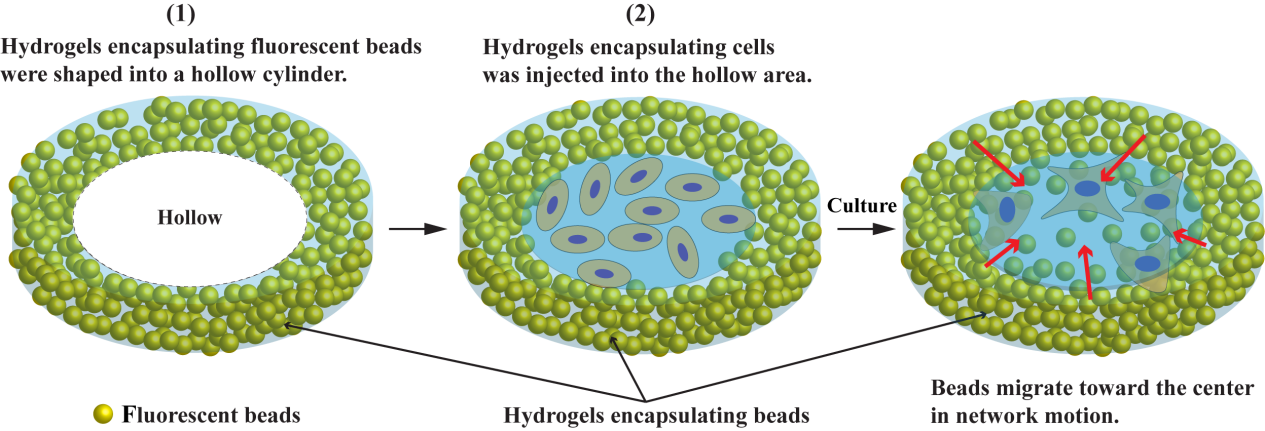


**Figure S14.**  Schematic diagram of fluorescent beads tracking experiment.

Table S1. **Sequences of primers for RT-qPCR.**

| **Gene** | **Forward primer** | **Reverse primer** |
| --- | --- | --- |
| **Human-Gapdh** | GGAGCGAGATCCCTCCAAAAT | GGCTGTTGTCATACTTCTCATGG |
| **Human-Yap1** | TAGCCCTGCGTAGCCAGTTA | TCATGCTTAGTCCACTGTCTGT |
| **Human-ITGβ1** | CAAGAGAGCTGAAGACTATCCCA | TGAAGTCCGAAGTAATCCTCCT |
| **Human-N-cadherin** | TGCGGTACAGTGTAACTGGG | GAAACCGGGCTATCTGCTCG |
| **Mouse-Gapdh** | AGGTCGGTGTGAACGGATTTG | TGTAGACCATGTAGTTGAGGTCA |
| **Mouse-Yap1** | TGAGATCCCTGATGATGTACCAC | TGTTGTTGTCTGATCGTTGTGAT |
| **Mouse-ITGβ1** | ATGCCAAATCTTGCGGAGAAT | TTTGCTGCGATTGGTGACATT |
| **Mouse-Rac1** | GAGACGGAGCTGTTGGTAAAA | ATAGGCCCAGATTCACTGGTT |
| **CD86** | GGTTCTGTACGAGCACTATT | TAGAGTCCAGTTGTTCCTGTC |
| **iNOS** | CAGACACATACTTTATGCCACC | GTCATGTTTGCCGTCACTC |
| **TNF-α** | CTCTTCTCATTCCTGCTTGTG | GGGAACTTCTCATCCCTTTG |
| **IL-6** | GTCAATTCCAGAAACCGCTAT | GTGGTTGTCACCAGCATCA |
| **IL-1β** | CCTGTTCTTTGAAGTTGACGG | GCTGGATGCTCTCATCAGGA |
| **CD163** | TGATGATAACTGGGATCTCCG | CCTCACTGGCATTAACTCG |
| **CD206** | TGAGGGAAGCGAGAGATTATG | CCAGGTTAAAGCAGACTTGG |
